# Genomic analyses reveal two distinct lineages of *Corynebacterium ulcerans* strains

**DOI:** 10.1016/j.nmni.2018.05.005

**Published:** 2018-05-25

**Authors:** R. Subedi, V. Kolodkina, I.C. Sutcliffe, L. Simpson-Louredo, R. Hirata, L. Titov, A.L. Mattos-Guaraldi, A. Burkovski, V. Sangal

**Affiliations:** 1) Faculty of Health and Life Sciences, Northumbria University, Newcastle upon Tyne, England, UK; 2) Republican Research and Practical Centre for Epidemiology and Microbiology, Minsk, Belarus; 3) Laboratory of Diphtheria and Corynebacteria of Clinical Relevance-LDCIC, Faculty of Medical Sciences, Rio de Janeiro State University, Rio de Janeiro, Brazil; 4) Mikrobiologie, Friedrich-Alexander-Universität Erlangen-Nürnberg, Germany

**Keywords:** *Corynebacterium ulcerans*, diphtheria, nontoxigenic, sore throat, toxigenic, vaccine, virulence, zoonotic

## Abstract

*Corynebacterium ulcerans* is an important zoonotic pathogen which is causing diphtheria-like disease in humans globally. In this study, the genomes of three recently isolated *C. ulcerans* strains, 4940, 2590 and BR-AD 2649, respectively from an asymptomatic carrier, a patient with pharyngitis and a canine host, were sequenced to investigate their virulence potential. A comparative analysis was performed including the published genome sequences of 16 other *C. ulcerans* isolates. *C. ulcerans* strains belong to two lineages; 13 strains are grouped together in lineage 1, and six strains comprise lineage 2. Consistent with the zoonotic nature of *C. ulcerans* infections, isolates from both the human and canine hosts clustered in both the lineages. Most of the strains possessed *spaDEF* and *spaBC* gene clusters along with the virulence genes *cpp*, *pld*, *cwlH*, *nanH*, *rpfI*, *tspA* and *vsp1*. The gene encoding Shiga-like toxin was only present in one strain, and 11 strains carried the *tox* gene encoding the diphtheria-like toxin. However, none of strains 4940, 2590 and BR-AD 2649 carried any toxin genes. These strains varied in the number of prophages in their genomes, which suggests that they play an important role in introducing diversity in *C. ulcerans.* The pan-genomic analyses revealed a variation in the number of membrane-associated and secreted proteins that may contribute to the variation in pathogenicity among different strains.

## Introduction

*Corynebacterium ulcerans* has emerged as an important zoonotic pathogen causing diphtheria-like infections in humans [1]. An increasing number of cases of *C. ulcerans* infection have been reported from many countries including Brazil, Germany, Italy and the United Kingdom [2], [3], [4], [5], [6]. Interestingly, these cases are more common in industrialized countries than in developing nations [1]. *C. ulcerans* is asymptomatically carried by a wide range of animals, which serve as a source of transmission to humans [1], [7].

Diphtheria-like *C. ulcerans* infections are caused by toxigenic strains carrying a *tox* gene on a lysogenizing corynephage [3], [8], [9]; however, the *tox* gene was also found to be present on a pathogenicity island in some strains [10]. Nontoxigenic strains that lack the *tox* gene and nontoxigenic *tox* gene–bearing *C. ulcerans* strains have also been isolated from animals and humans [11], [12], [13], [14]. In nontoxigenic *tox* gene–bearing strains, the *tox* gene is inactive as a result of frameshift mutations, but they may genetically revert to active toxin production [15]. The virulence potential has been found to vary among different *C. ulcerans* strains [12]. Several genes encoding virulence associated proteins such as phospholipase D (Pld), neuraminidase H (NanH), corynebacterial protease (CP40), venom serine protease (Vsp1 and Vsp2), ribosomal-binding protein (Rbp, similar to Shiga-like toxin) and adhesive surface pili are present in different *C. ulcerans* strains [3], [9], [16]. Variations in virulence may depend on the differences in the virulence gene repertoire among individual strains [17], [18].

Multilocus sequence typing data revealed extensive genetic diversity within *C. ulcerans* [19], [20], but the genome sequences of only 16 strains are publicly available (Supplementary Table S1). As a step towards characterizing more genomic diversity, we sequenced the genomes of three *C. ulcerans* strains. One strain was isolated from an asymptomatic human carrier in Belarus and two strains were isolated from Brazil, one from a patient with pharyngitis and the other from an asymptomatic dog. The genome sequences were compared with the publically available genome sequences of 16 *C. ulcerans* strains (Table 1) to gain insight into their virulence potential.

**Table 1:** Details of genome assemblies of *Corynebacterium ulcerans* strains

Detail	Strain		
	4940	2590	BR-AD 2649
Size of assembly (bp)	2 419 371	2 501 366	2 541 476
No. of contigs	21	10	17
Average coverage	302×	53×	94×
N50 (bp)	244 547	394 989	283 966
Average GC content (mol%)	53.3	53.3	53.3
No. of coding sequences	2175	2267	2310
No. of transfer RNA sequences	50	51	52

## Materials and methods

### Bacteria strains

*C. ulcerans* strain 4940 was isolated from an asymptomatic carrier with suspected contact with a diphtheria patient in the Minsk region of Belarus in 2009. Two strains, 2590 and BR-AD 2649, were isolated in Brazil, the former from a patient with pharyngitis and latter from an asymptomatic dog, in 2014 and 2015, respectively. Canine isolates of *C. ulcerans* are of interest for understanding the zoonotic relationship between human isolates and those carried by companion animals [21].

### Genome sequencing and assembly

The bacterial strains were cultured in 5 mL Brain–Heart Infusion broth (Oxoid, UK) and were incubated overnight at 37°C in a shaking incubator. Genomic DNA was extracted from 2 mL cultures using the UltraClean Microbial DNA Isolation Kit (MoBio, USA) and then sequenced on an Illumina MiSeq instrument (Illumina, USA). Paired-end reads were assembled using SPAdes 3.9.0 [22]. The draft assemblies were submitted to GenBank and are publicly available (Table 1; Supplementary Table S1).

### Comparative genomic analyses

The genome sequences of 16 previously published or publically available *C. ulcerans* strains were obtained from GenBank for comparative analyses (Supplementary Table S1). All genome sequences were annotated using Prokka [23] and were compared using Roary [24], [25]. A maximum-likelihood tree was constructed from the core genomic sequence alignment using IQ-Tree [26] with 100 000 ultrafast bootstraps and 100 000 SH-aLRT tests. This tree was visualized using the Interactive Tree of Life [27] and was rerooted on the longest branch. The prophage sequences in unannotated nucleotide sequences of draft genomes were identified using PHASTER [28]. The known prophage sequences ΦCULC809I, ΦCULC22I, ΦCULC22II, ΦCULC22III, ΦCULC22IV and ΦCULC0102-I were also searched in the draft assemblies of strains 4940, 2590 and BR-AD 2649 by nucleotide BLAST searches [29]. The presence or absence of previously reported virulence genes and *Spa* gene clusters [3], [16] in the genome sequences of strains 4940, 2590 and BR-AD 2649 were confirmed by protein BLAST searches [29], [30].

### Proteome prediction

Pan-genomic protein sequences were analysed to identify membrane-associated and secreted proteins using a previously published approach [18]. Briefly, signal peptides were identified by using the Phobius [31] and SignalP 4.1 web servers [32]. Lipoproteins were predicted using LipoP 1.0 [33] and PRED_Lipo [34]. Transmembrane domains were predicted using TMHHM 2.0 [35] and Phobius [31]. Cell wall–anchored proteins with LPXTG motif were predicted using the CW-PRED web server [36].

Proteins were assigned as secreted proteins if signal peptides were detected by both SignalP 4.1 and Phobius. Secreted proteins with a ‘lipobox’ detected by LipoP 1.0 and PRED_LIPO were assigned as lipoproteins. If lipoproteins were only detected by one of the programmes, sequences were analysed at the DOLOP web server to identify the ‘lipobox’ [37]. Proteins with transmembrane domains predicted by Phobius and TMHMM 2.0 were defined as membrane-associated proteins. Proteins with a signal peptide or transmembrane domains identified by a single prediction programme were assigned as ambiguous. Cell wall–anchored proteins generally have an N-terminal signal peptide and a membrane-spanning domain at the C terminal that follows the LPXTG motif [38]. Therefore, proteins with a C-terminal LPXTG motif and transmembrane domain but without any predicted signal peptide were scored as ambiguous, and the proteins where N-terminal signal peptide was detected by a single programme were manually inspected.

## Results and discussion

### Genomic features and prophage-like sequences in *C. ulcerans* strains

*C. ulcerans* strains 4940, 2590 and BR-AD 2649 were isolated between 2009 and 2015 from diverse sources. The size of the genome assemblies varied between 2.4 and 2.5 Mb, and the GC content of 53.3 mol% for all three; these values are comparable with the genomic characteristics of *C. ulcerans* strains in previous studies (Supplementary Table S1) [9], [10], [16]. The number of genes varied between 2175 for strain 4940 to 2310 for strain BR-AD 2649 (Table 1). The genome sequences of *C. ulcerans* strains 4940, 2590 and BR-AD 2649 are available from the GenBank database with the accession numbers LSWN00000000, MPSS00000000 and MPST00000000, respectively.

The genomes of *C. ulcerans* strains have been characterized by the presence of multiple prophages that are an important source of genomic plasticity in this pathogen [9], [10], [16]. Therefore, we searched the genomes of strains 4940, 2590, BR-AD 2649 using PHASTER [28] and identified multiple incomplete prophage sequences, probably due to the draft status of the genomes. The GC content of these predicted prophage sequences differed from the average GC content of 53.3 mol% of *C. ulcerans* genomes (Table 2).

**Table 2:** Predicted prophage sequences in genome sequences of *Corynebacterium ulcerans* strains

Strain	Prophage	Contig	Start	End	Size (kb)	No. of coding sequences	GC (mol%)
4940	I	5	3130	11 798	8.6	11	55.37
2590	I	1	735 327	74 4263	8.9	11	51.89
2590	II	2	388 519	419 587	31	12	50.11
2590	III	5	72 847	89 434	16.5	16	56.02
BR-AD 2649	I	1	238	8954	8.7	6	54.33
BR-AD 2649	II	2	179 366	187 941	8.5	11	55.42
BR-AD 2649	III	6	183 548	200 262	16.7	34	52.77
BR-AD 2649	IV	7	117 214	126 099	8.8	7	50.36
BR-AD 2649	V	14	3469	10 391	6.9	6	51.70

A BLAST search for the known prophage sequences of ΦCULC809I, ΦCULC22I, ΦCULC22II, ΦCULC22III, ΦCULC22IV and ΦCULC0102-I revealed an absence of all these prophages in strain 4940. Only one partial prophage, 8.6 kb in size, was predicted in the genome of this strain, which encompassed 11 genes, six encoding hypothetical proteins and five phage-associated proteins including RNA polymerase sigma factor, chaperonin GroEL and YgjD. According to the nucleotide BLAST searches, this region is also present in the published sequences of strains 809 and BR-AD 22 but was not identified as a prophage-associated region. The GC content of this region is 55.42 mol%, which is higher than the average GC content of the strain 4940 genome. In addition, PHASTER reported significant similarities among some of these genes and genes on previously reported phages in other species (data not shown). Therefore, it may be a prophage that was not detected previously.

Three incomplete prophages were identified in the draft assembly of strain 2590 (Table 2). The prophage on contig 1 is predicted to have 11 genes, eight encoding hypothetical or uncharacterized proteins and three genes coding for cytochrome *c* oxidase subunit I, putative ribonucleotide reductase and ribonucleotide reductase stimulatory protein. This region is present in strains 809 and BR-AD 22 (99% sequence similarity) but is not identified as prophage associated. The second prophage is approximately 31 kb in size and is integrated between *attL* and *attR* sites on contig 2. This region carries 12 genes, mostly encoding hypothetical proteins, and shows partial similarity with other *C. ulcerans* genomes (27–38% of the sequence with 96–99% identity). Therefore, this phage seems to be novel to this isolate. The third predicted prophage on contig 5 is similar to ΦCULC22IV of strain BR-AD 22 (>97% sequence identity).

Five prophages were predicted in the genome of BR-AD 2649, on contigs 1, 2, 6, 7 and 14. The sequences of prophages ΦCULC809I and ΦCULC22I showed significant similarity with the incomplete phage sequences on contigs 1 and 14 and additional small contigs 15 and 17 in strain BR-AD 2649. Prophages ΦCULC809I and ΦCULC22I are similar to each other in both size and gene content [16]. The prophage on contig 2 is similar to the phage predicted in strain 4940 in its size, GC content and gene content. The prophage sequence predicted on contig 6 has significant similarity with the genome of strain FRC58, isolated from the bronchitic aspiration of a patient in France [39]. The putative prophage sequences on contig 7 appear to be novel. Therefore, consistent with previous genomic studies, prophage-like sequences introduce significant diversity among *C. ulcerans* strains [9], [10], [16]. None of the three strains possessed a phage similar to the *tox* gene bearing phage ΦCULC0102-I [9].

**Fig. 1:**
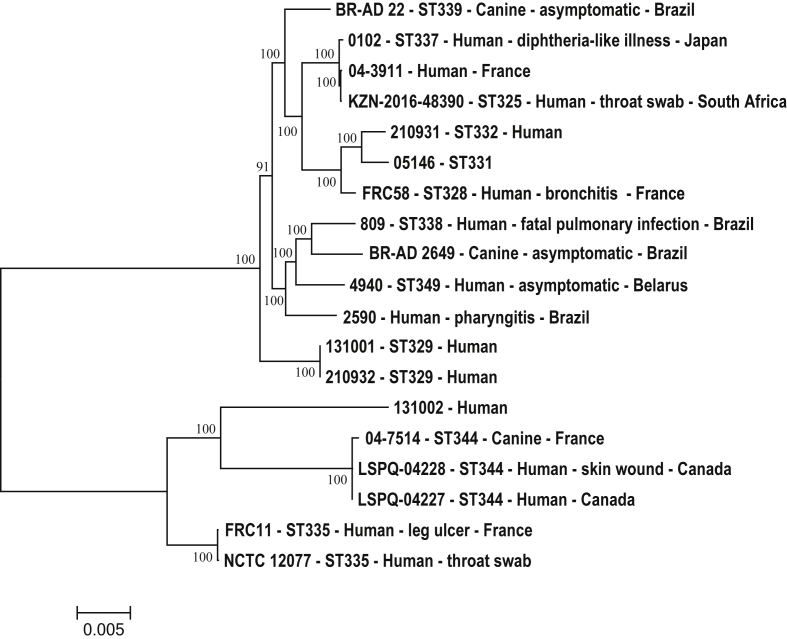
Maximum-likelihood tree derived from concatenated nucleotide-sequenced alignment of core genome. Scale bar represents nucleotide substitutions per nucleotide site. Strain designations are followed by sequence type (ST) designations, clinical information and country of isolation when this information was available.

Although some of the predicted prophage sequences are novel to strains 2590 and BR-AD 2649, each of these strains has at least one prophage that was previously reported in other Brazilian strains [16]. This may suggest that some corynebacteriophages are potentially more prevalent in certain geographic regions and lysogenizing *C. ulcerans* strains locally. Strain 809 was isolated from a patient with fatal pulmonary infection and BR-AD 22 from an asymptomatic dog [16]. It is interesting that ΦCULC22IV, from a canine isolate, is similar to the phage present in strain 2590, which was isolated from a patient with pharyngitis. The core genome phylogeny suggests that BR-AD 22 and 2590 are quite distant from each other (Fig. 1), and it is possible that the same bacteriophage independently lysogenized each strain.

### Virulence potential of *C. ulcerans* strains

Surface pili are responsible for adhesion and invasion to the host cells, which play an important role in the virulence of pathogenic bacteria [40], [41]. A variation in the number and organization of pilus gene clusters was found to correlate with the adhesive and invasive properties of *Corynebacterium diphtheriae* isolates [18], [42]. Two pilus gene clusters, namely *spaDEF* and *spaBC*, have been identified in *C. ulcerans* genomes [16], and both of them are present in the three strains sequenced in this study. The *spaDEF* cluster is composed of five genes including *spaD*, *spaE* and *spaF* encoding the major pilin subunit, minor subunit and the tip protein, respectively, and two sortase genes, *srtB* and *srtC*, which are responsible for the pilus assembly (Supplementary Fig. S1). The organization of this gene cluster is conserved among the three strains, except that in strain 4940 the *spaD* and *spaF* genes are each present as two smaller genes where the coding sequences are interrupted, creating a potential coding sequence for a secreted version of the N-terminal domain and a separate coding sequence encoding a putative wall anchored corresponding to the C-terminal domain. *spaDEF* pili interact with laryngeal epithelial cells in *C. diphtheriae* [41], [43]. The *spaBC* cluster has three genes: *spaB* encoding the minor pilin subunit, *spaC* encoding the tip protein and a sortase *srtA* (Supplementary Fig. S1). A major pilin subunit is absent from this cluster, and the interaction to pharyngeal epithelial cells was suggested to be through homodimeric or heterodimeric SpaB/SpaC proteins [16].

Other putative virulence genes, including *cpp*, *pld*, *cwlH*, *nanH*, *rpfI*, *tspA* and *vsp1* [3], [16], were present in all three strains (Table 3). However, similar to another canine isolate, BR-AD 22 [16], the *vsp2* gene was absent in strain BR-AD 2649. The genes encoding the Shiga-like toxin (*rbp*) and diphtheria-like toxin (*tox*) were absent in these *C. ulcerans* isolates (Table 3). Toxigenic *C. ulcerans* strains are often associated with fatal outcomes [16], [44]; however, nontoxigenic strains are equipped with other virulence genes and may still be able to cause severe invasive infections. These data do not show any clear variations in the virulence gene repertoire among the strains isolated from patients with disease and asymptomatic carriers. Our previous study showed that the same *C. diphtheriae* strains can cause diphtheria in some individuals and remain asymptomatic in others [17].

**Table 3:** Presence and absence of known virulence genes in *Corynebacterium ulcerans* strains

Gene	Function	No. of strains	4940	2590	BR-AD 2649
*cpp*	Corynebacterial protease CP40	19	4940_01651	2590_02130	BRAD2649_00957
*cwlH*^a^	Cell wall–associated hydrolase	19	4940_00472	2590_00418	BRAD2649_00367
*nanH*^a^	Sialidase precursor (neuraminidase H)	19	4940_01018	2590_00864	BRAD2649_00647
*pld*	Phospholipase D precursor	19	4940_01294	2590_01624	BRAD2649_01264
*rpfI*	Rpf-interacting protein	19	4940_00122	2590_00067	BRAD2649_01856
*spaB*	Surface-anchored protein	19	4940_01657	2590_02124	BRAD2649_00951
*spaC*	Surface-anchored protein	19	4940_01656	2590_02125	BRAD2649_00952
*spaD*^a^	Surface-anchored protein	18	4940_01627/ 4940_01628	2590_02003	BRAD2649_00993
*spaE*	Surface-anchored protein	19	4940_01625	2590_02001	BRAD2649_00995
*spaF*^a^	Surface-anchored protein	19	4940_01623/ 4940_01624	2590_02000	BRAD2649_00996
*tspA*	Trypsin-like serine protease	19	4940_01462	2590_01903	BRAD2649_01091
*vsp1*^a^	Venom serine protease	19	4940_02126	2590_00941	BRAD2649_00571
*vsp2*	Venom serine protease	14	4940_01640	2590_02141	—
*rbp*	Ribosome-binding protein	1	*—*	*—*	*—*
*tox*	Diphtheria-like toxin	11	*—*	*—*	*—*

a Genes annotated as multiple short coding sequences in some strains.

### Genomic diversity among *C. ulcerans* strains

*C. ulcerans* is genetically quite diverse, with two major clonal groups and multiple singleton sequence types (STs), based on multilocus sequence typing data [19], [20]. To investigate the diversity at the genomic level, the publicly available genome sequences of 16 *C. ulcerans* strains were included for a comparative analysis (Supplementary Table S1). A phylogenetic tree from the concatenated core genome (1405 genes) separated *C. ulcerans* into two lineages, one assembled with 13 strains and the other with six isolates (Fig. 1). Both the major clonal groups (eBG325 and eBG332) and some singleton STs (ST329, ST338, ST339 and ST349) were grouped in lineage 1 [19], [20], along with strains 04-3911, 2590 and BR-AD 2649, which could not be assigned an ST designation because of the presence of new alleles (Fig. 1). Lineage 2 includes isolates belonging to ST335 and ST344, as well as one strain, 131002, which was also not assigned an ST designation (Fig. 1). ST335 and ST344 were singletons in previous studies [19], [20].

Distinct phylogenetic groups were also previously reported on the basis of the analysis of genome-wide single nucleotide polymorphisms among nine *C. ulcerans* strains [10]. Four of the nine genome sequences (0102, 809, BR-AD 22 and FRC58) are also included in this study, and all of them belong to lineage 1 (Fig. 1). Both the lineages include strains from canine and human hosts, which is consistent with the zoonotic nature of *C. ulcerans* infections [1], [10]. Lineage 1 encompasses strains from Belarus, Brazil, France, Japan and South Africa, whereas most of the isolates in lineage 2 were isolated in Canada and France (Supplementary Table S1). However, more *C. ulcerans* strains need to be analysed to permit inference of any geographic association.

Most of the putative virulence genes are present in all 19 isolates, with the exception of *spaD*, which is absent from strain 04-3911, and *vsp2*, which is absent from five isolates (Table 3). The Shiga-like toxin gene (*rbp*) is only present in strain 809, whereas the *tox* gene encoding diphtheria-like toxin is more common, being present in 11 of the 19 isolates. The Rbp protein shows structural similarities, particularly the catalytic residues, to Shiga-like toxins SLT-1 and SLT-2 present in *Escherichia coli* [16]. Shiga-like toxins can cause severe damage to human organs, including vascular endothelial cells, intestine, kidneys and brain [45], [46]. Strain 809 was isolated from a fatal pulmonary infection in an elderly woman [47]. The patient was administered diphtheria antitoxin and was treated with different combinations of antibiotics, but she died of multiple organ failure [47]. Shiga-like toxins are quite unusual in *C. ulcerans* and may have contributed to the organ failure in this patient. The *rbp* gene is flanked by genes encoding phage integrase and transposase and a variation in the DNA G+C content (45.1 mol%) when compared to the genome (53.3 mol%), suggesting the acquisition of this gene by strain 809 by recombination [16]. However, the presence or absence of these virulence genes has no association with either of the lineages.

The pan-genome of the 19 *C. ulcerans* strains was found to be composed of 4120 genes, including 1405 core genes and 2715 accessory genes. Transmembrane domains were detected in 351 of the core proteins, 13 with additional signal peptides, and two were cell wall–anchored proteins (Supplementary Table S2). Eighty-two of the core proteins were predicted to be secreted, of which 46 were identified as putative lipoproteins. Sixty-six core proteins were scored as ambiguous because of a lack of consensus among the prediction tools. The accessory genome included 611 membrane-associated proteins, 65 with additional signal peptide features and 46 with an LPXTG motif (Supplementary Table S2). A total of 116 accessory proteins were secreted via sec-dependent secretory pathways (Supplementary Table S2). Membrane-associated and secreted proteins are important for host–pathogen interactions and virulence [18], [48], [49], [50]. Therefore, in addition to the variation in the virulence genes, the number of transmembrane, lipoprotein and secreted proteins may be responsible for the variation in their virulence characteristics. Indeed, a variation in the ability to cause arthritis in a mice model by different *C. ulcerans* strains was previously reported [12]. As mentioned earlier, prophages are the major source of diversity among these strains [16].

## Conclusion

*C. ulcerans* strains are genetically diverse and belong to two distinct lineages. Genomic analyses revealed variations in the proteins with transmembrane domains among different strains, including some genes involved in the synthesis of pili, which may affect their ability to adhere to the host cells. *C. ulcerans* strains have been reported to vary in the degree of pathogenesis, which may be caused by variations in the secreted proteins. The number of prophages varied among different strains, which is a major source of plasticity in *C. ulcerans* genomes. A majority of *C. ulcerans* strains possessed the *tox* gene, which is also present on a bacteriophage and is responsible for diphtheria-like infection in humans.

## Conflict of interest

None declared.
