# Supplementary material for: Genomic analyses reveal two distinct lineages of Corynebacterium ulcerans strains
Source: New Microbes New Infect. 2018 May 25;25:7–13. doi: 10.1016/j.nmni.2018.05.005 (PMC6038270; doi:10.1016/j.nmni.2018.05.005)
Supplement: Multimedia component 1 [file mmc1.pdf]

Supplementary Table 1. Details of strains and genomes analysed in this study

Cluster	Strain ID	Genome size (Mb)	GC (Mol%)	Host	Year	Country	Clinical information	Accession Numbers	Reference or genome release date
1	BR-AD 2649	2.54	53.3	Canine	2015	Brazil	asymptomatic	MPST00000000	This study
1	BR-AD 22	2.61	53.4	Canine	2010	Brazil	asymptomatic	NC_015683.1	(Trost et al., 2011)
1	0102	2.58	53.4	Human	2001	Japan	diphtheria-like illness	NC_018101.1	(Sekizuka et al., 2012)
1	04-3911	2.49	53.3	Human	2004	France		LGSX00000000.1	(Guimaraes et al., 2016a)
1	131001	2.48	53.3	Human				NZ_CP010818.1	14/09/2015
1	210931	2.51	53.3	Human				NZ_CP009583.1	22/10/2014
1	210932	2.48	53.3	Human				NZ_CP009500.1	(Viana et al., 2014)
1	2590	2.50	53.3	Human	2014	Brazil	pharyngitis	MPSS00000000	This study
1	4940	2.42	53.3	Human	2009	Belarus	asymptomatic	LSWN00000000	This study
1	809	2.50	53.3	Human	2000	Brazil	fatal pulmonary infection	NC_017317.1	(Trost et al., 2011)
1	FRC58	2.54	53.3	Human		France	bronchitis	NZ_CP011913.1	(Silva Ado et al., 2014)
1	KZN-2016-48390	2.54	53.4	Human		South Africa	throat swab	MIOS00000000.1	10/11/2016
1	05146	2.47	53.3					NZ_CP009716.1	28/10/2014
2	04-7514	2.50	53.5	Canine	2004	France		LJVH00000000.1	(Guimaraes et al., 2016b)
2	131002	2.43	53.4	Human				NZ_CP011095.1	03/04/2015
2	FRC11	2.44	53.4	Human		France	leg ulcer	NZ_CP009622	(Benevides Lde et al., 2015)
2	LSPQ-04227	2.43	53.4	Human	2004	Canada		JZUS00000000.1	(Domingo et al., 2015)
2	LSPQ-04228	2.44	53.4	Human	2013	Canada	skin wound	JZUT00000000.1	(Domingo et al., 2015)
2	NCTC 12077	2.62	53.4	Human			throat swab	AYUJ00000000.1	25/11/2013

References

Benevides Lde, J., Viana, M.V., Mariano, D.C., Rocha Fde, S., Bagano, P.C., Folador, E.L., Pereira, F.L., Dorella, F.A., Leal, C.A., Carvalho, A.F., Soares Sde, C., Carneiro, A., Ramos, R., Badell-Ocando, E., Guiso, N., Silva, A., Figueiredo, H., Azevedo, V., Guimaraes, L.C., 2015. Genome Sequence of *Corynebacterium ulcerans* Strain FRC11. Genome Announc 3.

Domingo, M.C., Fournier, E., Masse, C., Charest, H., Bernard, K., Cote, J.C., Tremblay, C., 2015. Draft Genome Sequences of Two Toxigenic *Corynebacterium ulcerans* Strains. Genome Announc 3.

Guimaraes, L.C., Viana, M.V., Benevides, L.J., Mariano, D.C., Veras, A.A., Sa, P.H., Rocha, F.S., Vilas Boas, P.C., Soares, S.C., Barbosa, M.S., Guiso, N., Badell, E., Carneiro, A.R., Azevedo, V., Ramos, R.T., Silva, A., 2016a. Draft Genome Sequence of *Corynebacterium ulcerans* Strain 04-3911, Isolated from Humans. Genome Announc 4.

Guimaraes, L.C., Viana, M.V., Benevides, L.J., Mariano, D.C., Veras, A.A., Sa, P.H., Rocha, F.S., Vilas Boas, P.C., Soares, S.C., Barbosa, M.S., Guiso, N., Badell, E., Carneiro, A.R., Azevedo, V., Ramos, R.T., Silva, A., 2016b. Draft Genome Sequence of Toxigenic *Corynebacterium ulcerans* Strain 04-7514, Isolated from a Dog in France. Genome Announc 4.

Sekizuka, T., Yamamoto, A., Komiya, T., Kenri, T., Takeuchi, F., Shibayama, K., Takahashi, M., Kuroda, M., Iwaki, M., 2012. *Corynebacterium ulcerans* 0102 carries the gene encoding diphtheria toxin on a prophage different from the *C. diphtheriae* NCTC 13129 prophage. BMC Microbiol 12, 72.

Silva Ado, S., Barauna, R.A., de Sa, P.C., das Gracas, D.A., Carneiro, A.R., Thouvenin, M., Azevedo, V., Badell, E., Guiso, N., da Silva, A.L., Ramos, R.T., 2014. Draft Genome Sequence of *Corynebacterium ulcerans* FRC58, Isolated from the Bronchitic Aspiration of a Patient in France. Genome Announc 2.

Trost, E., Al-Dilaimi, A., Papavasiliou, P., Schneider, J., Viehoever, P., Burkovski, A., Soares, S.C., Almeida, S.S., Dorella, F.A., Miyoshi, A., Azevedo, V., Schneider, M.P., Silva, A., Santos, C.S., Santos, L.S., Sabbadini, P., Dias, A.A., Hirata, R., Jr., Mattos-Guaraldi, A.L., Tauch, A., 2011. Comparative analysis of two complete *Corynebacterium ulcerans* genomes and detection of candidate virulence factors. BMC Genomics 12, 383.

Viana, M.V., de Jesus Benevides, L., Batista Mariano, D.C., de Souza Rocha, F., Bagano Vilas Boas, P.C., Folador, E.L., Pereira, F.L., Alves Dorella, F., Gomes Leal, C.A., Fiorini de Carvalho, A., Silva, A., de Castro Soares, S., Pereira Figueiredo, H.C., Azevedo, V., Guimaraes, L.C., 2014. Genome Sequence of *Corynebacterium ulcerans* Strain 210932. Genome Announc 2.
