# Supplementary material for: Genomic analyses reveal two distinct lineages of Corynebacterium ulcerans strains
Source: New Microbes New Infect. 2018 May 25;25:7–13. doi: 10.1016/j.nmni.2018.05.005 (PMC6038270; doi:10.1016/j.nmni.2018.05.005)
Supplement: Multimedia component 2 [file mmc2.pdf]

Supplementary Table 2. A list of proteins identified with signal peptides, transmembrane domains and LPXTG motifs

S.No.	LocusTags	Annotation	Type	SignalP	Phobius-SP	LipoP	PREDLipo	DOLOP	Phobius-TM	TMHMM	CW-PRED	Final
1	809_00046	Serine/threonine-protein kinase PknA	Core						1			Amb
2	809_00047	Penicillin-binding protein A	Core		Y					1		Amb
3	809_00066	Oligo-1,6-glucosidase 1	Core						1			Amb
4	809_00148	hypothetical protein	Core		Y					1		Amb
5	809_00194	prephenate dehydrogenase	Core		Y							Amb
6	809_00212	2-hydroxy-3-oxopropionate reductase	Core		Y							Amb
7	809_00239	Catalase	Core									Amb
8	809_00249	Na(+)/H(+) antiporter subunit E	Core		Y							Amb
9	809_00258	phosphodiesterase YaeI	Core		Y					1		Amb
10	809_00311	Formate acetyltransferase	Core									Amb
11	809_00319	Signal-transduction histidine kinase senX3	Core		Y					1		Amb
12	809_00330	Phosphoserine phosphatase	Core						1			Amb
13	809_00339	Protoporphyrinogen oxidase	Core		Y							Amb
14	809_00377	N-ethylmaleimide reductase	Core		Y							Amb
15	809_00453	Adenylate kinase	Core		Y							Amb
16	809_00460	30S ribosomal protein S11	Core									Amb
17	809_00475	Phosphoglucosamine mutase	Core		Y							Amb
18	809_00491	hypothetical protein	Core							1		Amb
19	809_00556	hypothetical protein	Core									Amb
20	809_00557	putative propionyl-CoA carboxylase beta chain 5	Core							1		Amb
21	809_00603	Cys-tRNA(Pro)/Cys-tRNA(Cys) deacylase YbaK	Core		Y							Amb
22	809_00622	Lon protease 2	Core		Y							Amb
23	809_00756	Resuscitation-promoting factor Rpf2 precursor	Core		Y					1		Amb
24	809_00787	Transposon Tn10 TetC protein	Core						1			Amb
25	809_00791	hypothetical protein	Core		Y					1		Amb
26	809_00866	hypothetical protein	Core		Y					1		Amb
27	809_00876	sec-independent translocase	Core						1			Amb
28	809_00883	hypothetical protein	Core						3			Amb
29	809_00898	hypothetical protein	Core		Y					1		Amb
30	809_00914	Lactate utilization protein A	Core		Y							Amb
31	809_00950	tRNA-specific 2-thiouridylase MnmA	Core		Y							Amb
32	809_01070	Copper transporter MctB precursor	Core		Y					1		Amb
33	809_01075	ATP-dependent dethiobiotin synthetase BioD	Core		Y							Amb
34	809_01108	6-phosphogluconate dehydrogenase, NADP(+)-dependent, decarboxylating	Core		Y							Amb
35	809_01122	hypothetical protein	Core		Y					1		Amb
36	809_01129	Sulfite reductase [ferredoxin]	Core	Y								Amb
37	809_01150	putative HTH-type transcriptional regulator YddM	Core									Amb
38	809_01179	Modulator of FtsH protease HflK	Core		Y					1		Amb
39	809_01378	UDP-glucose 4-epimerase	Core		Y							Amb
40	809_01398	FMN reductase (NADPH)	Core		Y							Amb
41	809_01442	hypothetical protein	Core				Lipoprotein		1			Amb
42	809_01451	Magnesium-chelatase 60 kDa subunit	Core		Y							Amb
43	809_01538	hypothetical protein	Core							1		Amb
44	809_01548	hypothetical protein	Core				Lipoprotein					Amb
45	809_01559	Cell division protein FtsQ	Core		Y					1		Amb
46	809_01563	UDP-N-acetylmuramoylalanine--D-glutamate ligase	Core						1			Amb
47	809_01566	UDP-N-acetylmuramoyl-L-alanyl-D-glutamate--2,6-diaminopimelate ligase	Core		Y							Amb
48	809_01594	Putative asparagine synthetase [glutamine-hydrolyzing]	Core		Y							Amb
49	809_01667	hypothetical protein	Core						1			Amb
50	809_01757	hypothetical protein	Core		Y					1		Amb
51	809_01785	3-oxoacyl-[acyl-carrier-protein] reductase FabG	Core						1			Amb
52	809_01808	Ribonuclease Z	Core		Y							Amb
53	809_01872	hypothetical protein	Core		Y					1		Amb
54	809_01882	Ribonucleoside-diphosphate reductase subunit beta nrdF2	Core						1			Amb
55	809_01900	Bacterial regulatory proteins, tetR family	Core		Y							Amb
56	809_01918	2-C-methyl-D-erythritol 4-phosphate cytidylyltransferase	Core		Y							Amb
57	809_01927	hypothetical protein	Core									Amb
58	809_01935	putative peptidase precursor	Core	Y								Amb
59	809_02015	hypothetical protein	Core		Y							Amb
60	809_02029	hypothetical protein	Core		Y					1		Amb
61	809_02061	Chaperone protein DnaJ	Core									Amb
62	809_02113	putative methyltransferase	Core						2			Amb
63	809_02132	undecaprenyl pyrophosphate phosphatase	Core						1			Amb
64	809_02177	hypothetical protein	Core	Y								Amb

65	809_02105	VanW like protein	Core		Y					1	Amb
66	809_00361	hypothetical protein	Core			SpII		No	2	2	Amb
67	809_00381	Periplasmic zinc-binding protein TroA precursor	Core		Y		Lipoprotein	Lipobox			Sec-Lipo
68	809_00880	hypothetical protein	Core		Y	SpII		Lipobox			Sec-Lipo
69	809_01613	Fe(3+)-citrate-binding protein YfmC precursor	Core	Y			Lipoprotein	Lipobox	1		Sec-Lipo
70	809_00078	hypothetical protein	Core	Y	Y	SpII	Lipoprotein				Sec-Lipo
71	809_00101	hypothetical protein	Core	Y	Y	SpII	Lipoprotein				Sec-Lipo
72	809_00150	Glycine betaine/carnitine/choline-binding protein OpuCC precursor	Core		Y	SpII	Lipoprotein				Sec-Lipo
73	809_00343	Thiol-disulfide oxidoreductase ResA	Core	Y	Y	SpII	Lipoprotein				Sec-Lipo
74	809_00426	putative monoacyl phosphatidylinositol tetramannoside-binding protein LpqW precursor	Core	Y	Y	SpII	Lipoprotein				Sec-Lipo
75	809_00445	Maltose/maltodextrin-binding protein precursor	Core	Y	Y	SpII	Lipoprotein				Sec-Lipo
76	809_00501	hypothetical protein	Core	Y	Y	SpII	Lipoprotein				Sec-Lipo
77	809_00505	D-methionine-binding lipoprotein MetQ precursor	Core		Y	SpII	Lipoprotein				Sec-Lipo
78	809_00506	D-methionine-binding lipoprotein MetQ precursor	Core	Y	Y	SpII	Lipoprotein				Sec-Lipo
79	809_00509	putative periplasmic iron-binding protein precursor	Core	Y	Y	SpII	Lipoprotein				Sec-Lipo
80	809_00521	Hemin-binding periplasmic protein HmuT precursor	Core	Y	Y	SpII	Lipoprotein				Sec-Lipo
81	809_00573	Trehalose-binding lipoprotein LpqY precursor	Core	Y	Y	SpII	Lipoprotein				Sec-Lipo
82	809_00591	Lipoprotein LpqB precursor	Core	Y	Y	SpII	Lipoprotein				Sec-Lipo
83	809_00623	hypothetical protein	Core	Y	Y	SpII	Lipoprotein				Sec-Lipo
84	809_00653	putative ABC transporter solute-binding protein YclQ precursor	Core	Y	Y	SpII	Lipoprotein				Sec-Lipo
85	809_00813	hypothetical protein	Core	Y	Y	SpII	Lipoprotein				Sec-Lipo
86	809_00839	Oligopeptide-binding protein OppA precursor	Core	Y	Y	SpII	Lipoprotein				Sec-Lipo
87	809_00962	Vitamin B12-binding protein precursor	Core	Y	Y	SpII	Lipoprotein				Sec-Lipo
88	809_00982	hypothetical protein	Core	Y	Y	SpII	Lipoprotein				Sec-Lipo
89	809_01004	Oligopeptide-binding protein AppA precursor	Core	Y	Y	SpII	Lipoprotein				Sec-Lipo
90	809_01025	Glutamine-binding periplasmic protein precursor	Core	Y	Y	SpII	Lipoprotein				Sec-Lipo
91	809_01344	hypothetical protein	Core	Y	Y	SpII	Lipoprotein				Sec-Lipo
92	809_01549	hypothetical protein	Core	Y	Y	SpII	Lipoprotein				Sec-Lipo
93	809_01660	hypothetical protein	Core	Y	Y	SpII	Lipoprotein				Sec-Lipo
94	809_01694	hypothetical protein	Core	Y	Y	SpII	Lipoprotein				Sec-Lipo
95	809_01702	Heme-binding protein A precursor	Core	Y	Y	SpII	Lipoprotein				Sec-Lipo
96	809_01789	Putative L,D-transpeptidase LppS precursor	Core	Y	Y	SpII	Lipoprotein				Sec-Lipo
97	809_01870	Phosphate-binding protein PstS 3 precursor	Core	Y	Y	SpII	Lipoprotein				Sec-Lipo
98	809_01911	hypothetical protein	Core	Y	Y	SpII	Lipoprotein				Sec-Lipo
99	809_01920	hypothetical protein	Core	Y	Y	SpII	Lipoprotein				Sec-Lipo
100	809_02071	Oligopeptide-binding protein AppA precursor	Core	Y	Y	SpII	Lipoprotein				Sec-Lipo
101	809_02079	corrinoid ABC transporter substrate-binding protein	Core	Y	Y	SpII	Lipoprotein				Sec-Lipo
102	809_02106	Beta-hexosaminidase	Core	Y	Y	SpII	Lipoprotein				Sec-Lipo
103	809_02126	hypothetical protein	Core	Y	Y	SpII	Lipoprotein			1	Sec-Lipo
104	809_02136	Glycerophosphoryl diester phosphodiesterase precursor	Core	Y	Y	SpII	Lipoprotein				Sec-Lipo
105	809_02141	Fe(3+)-citrate-binding protein YfmC precursor	Core	Y	Y	SpII	Lipoprotein				Sec-Lipo
106	809_00630	hypothetical protein	Core	Y	Y		Lipoprotein	Lipobox		1	Sec-Lipo
107	809_00129	hypothetical protein	Core	Y	Y	SpII		Lipobox			Sec-Lipo
108	809_00435	L-asparaginase precursor	Core	Y	Y	SpII	Lipoprotein				Sec-Lipo
109	809_00651	Carbonic anhydrase 1	Core	Y	Y	SpII	Lipoprotein				Sec-Lipo
110	809_00696	Oligopeptide-binding protein AppA precursor	Core	Y	Y	SpII	Lipoprotein				Sec-Lipo
111	809_00784	Multicopper oxidase mco	Core		Y	SpII	Lipoprotein				Sec-Lipo
112	809_00161	Periplasmic zinc-binding protein TroA precursor	Core	Y	Y		Lipoprotein	Lipobox			Sec-Lipo
113	809_00851	putative monoacyl phosphatidylinositol tetramannoside-binding protein LpqW precursor	Core	Y	Y	SpII		No			Sec-SpI
114	809_00992	Esterase PHB depolymerase	Core	Y	Y	SpII		No			Sec-SpI
115	809_01992	ABC transporter glutamine-binding protein GlnH precursor	Core	Y	Y	SpII		No			Sec-SpI
116	809_00039	Phospholipase D precursor	Core	Y	Y					1	Sec-SpI
117	809_00103	Lysozyme M1 precursor	Core	Y	Y						Sec-SpI
118	809_00165	Periplasmic zinc-binding protein TroA precursor	Core	Y	Y						Sec-SpI
119	809_00260	Penicillin-binding protein 1F	Core	Y	Y					1	Sec-SpI
120	809_00281	hypothetical protein	Core	Y	Y					1	Sec-SpI
121	809_00292	hypothetical protein	Core	Y	Y						Sec-SpI
122	809_00328	Disulfide bond formation protein D precursor	Core		Y					1	Sec-SpI
123	809_00437	Major phosphate-irrepressible acid phosphatase precursor	Core	Y	Y						Sec-SpI
124	809_00483	hypothetical protein	Core	Y	Y					1	Sec-SpI
125	809_00608	hypothetical protein	Core	Y	Y					1	Sec-SpI
126	809_00641	hypothetical protein	Core	Y	Y					1	Sec-SpI
127	809_00761	hypothetical protein	Core	Y	Y					1	Sec-SpI
128	809_00846	putative peptidase precursor	Core	Y	Y					1	Sec-SpI
129	809_00849	hypothetical protein	Core	Y	Y					1	Sec-SpI
130	809_00909	hypothetical protein	Core	Y	Y					1	Sec-SpI
131	809_00971	hypothetical protein	Core	Y	Y					1	Sec-SpI

132	809_00995	Cutinase	Core	Y	Y		1	Sec-SpI
133	809_01184	Peptidoglycan endopeptidase RipA precursor	Core	Y	Y		1	Sec-SpI
134	809_01585	putative endopeptidase precursor	Core	Y	Y		1	Sec-SpI
135	809_01586	putative endopeptidase precursor	Core	Y	Y			Sec-SpI
136	809_01629	hypothetical protein	Core	Y	Y		1	Sec-SpI
137	809_01782	hypothetical protein	Core	Y	Y	1		Sec-SpI
138	809_01901	Diacylglycerol acyltransferase/mycolyltransferase Ag85B precursor	Core	Y	Y			Sec-SpI
139	809_01954	D-alanyl-D-alanine carboxypeptidase DacC precursor	Core	Y	Y		1	Sec-SpI
140	809_01958	putative inactive lipase/MT1628	Core	Y	Y			Sec-SpI
141	809_02017	hypothetical protein	Core	Y	Y			Sec-SpI
142	809_02058	hypothetical protein	Core	Y	Y		1	Sec-SpI
143	809_02059	Endo-beta-N-acetylglucosaminidase F2 precursor	Core	Y	Y		1	Sec-SpI
144	809_02125	Cutinase	Core	Y	Y		1	Sec-SpI
145	809_02127	Diacylglycerol acyltransferase/mycolyltransferase Ag85B precursor	Core	Y	Y		1	Sec-SpI
146	809_02129	Diacylglycerol acyltransferase/mycolyltransferase Ag85C precursor	Core	Y	Y		1	Sec-SpI
147	809_02148	putative membrane protein/MT3943	Core	Y	Y		1	Sec-SpI
148	809_00254	hypothetical protein	Core	Y	Y			Sec-SpI
149	809_00042	Ferric enterobactin transport system permease protein FepD	Core			9	9	TM
150	809_00044	Cell division protein CrgA	Core			2	2	TM
151	809_00045	Serine/threonine-protein kinase PknB	Core			1	1	TM
152	809_00048	Lipid II flippase FtsW	Core			12	12	TM
153	809_00050	FHA domain-containing protein FhaB	Core			1	1	TM
154	809_00058	CAAX amino terminal protease self- immunity	Core			6	6	TM
155	809_00060	hypothetical protein	Core			2	2	TM
156	809_00064	Hemin transport system permease protein HmuU	Core		Y	9	9	TM
157	809_00065	Magnesium transporter MgtE	Core			5	5	TM
158	809_00069	hypothetical protein	Core		Y	2	3	TM
159	809_00071	sensory histidine kinase UhpB	Core		Y	4	4	TM
160	809_00076	Anti-sigma-K factor rskA	Core			1	1	TM
161	809_00082	Glycine betaine/carnitine/choline transport system permease protein OpuCB	Core		Y	3	4	TM
162	809_00083	Carnitine transport permease protein OpuCD	Core			6	5	TM
163	809_00105	VIT family protein	Core			5	5	TM
164	809_00122	hypothetical protein	Core			6	5	TM
165	809_00126	hypothetical protein	Core			4	4	TM
166	809_00128	Putative ammonia monooxygenase	Core			10	10	TM
167	809_00130	hypothetical protein	Core			7	7	TM
168	809_00132	Ascorbate-specific permease IIC component UlaA	Core			11	11	TM
169	809_00145	putative glycerol uptake facilitator protein	Core			6	6	TM
170	809_00149	hypothetical protein	Core			6	8	TM
171	809_00151	putative arabinosyltransferase C	Core			13	13	TM
172	809_00152	Arabinofuranosyltransferase AftA	Core			13	10	TM
173	809_00156	hypothetical protein	Core			3	2	TM
174	809_00159	GtrA-like protein	Core			3	3	TM
175	809_00163	Manganese transport system membrane protein MntB	Core			7	8	TM
176	809_00164	Manganese transport system membrane protein MntB	Core			9	9	TM
177	809_00168	Teichoic acid translocation permease protein TagG	Core			6	6	TM
178	809_00178	PTS system glucose-specific EIICBA component	Core			10	10	TM
179	809_00201	Gnt-II system L-idonate transporter	Core			13	13	TM
180	809_00211	Inner membrane permease YgbN	Core			12	11	TM
181	809_00231	hypothetical protein	Core			9	10	TM
182	809_00232	hypothetical protein	Core		Y	7	8	TM
183	809_00234	ABC-2 family transporter protein	Core			13	12	TM
184	809_00247	putative monovalent cation/H+ antiporter subunit G	Core			3	3	TM
185	809_00248	putative monovalent cation/H+ antiporter subunit F	Core			3	3	TM
186	809_00250	Na(+)/H(+) antiporter subunit D	Core			14	14	TM
187	809_00251	Na(+)/H(+) antiporter subunit C1	Core		Y	2	3	TM
188	809_00268	Thiol-disulfide oxidoreductase ResA	Core			1	1	TM
189	809_00270	Serine protease	Core		Y	3	3	TM
190	809_00272	hypothetical protein	Core			2	2	TM
191	809_00274	Phosphoserine phosphatase	Core			1	1	TM
192	809_00279	Bacterial type II secretion system protein F domain protein	Core		Y	1	1	TM
193	809_00280	hypothetical protein	Core			1	1	TM
194	809_00282	hypothetical protein	Core			1	1	TM
195	809_00287	Adenylate cyclase 2	Core			6	6	TM
196	809_00302	Succinate dehydrogenase/Fumarate reductase transmembrane subunit	Core			5	5	TM
197	809_00305	hypothetical protein	Core			2	2	TM
198	809_00306	hypothetical protein	Core			3	3	TM

199	809_00309	hypothetical protein	Core			1	1	TM
200	809_00329	Cytochrome C biogenesis protein transmembrane region	Core			7	7	TM
201	809_00336	hypothetical protein	Core			4	4	TM
202	809_00337	putative copper-exporting P-type ATPase V	Core			7	7	TM
203	809_00344	Cytochrome C biogenesis protein transmembrane region	Core			6	6	TM
204	809_00345	Cytochrome c biogenesis protein Ccs1	Core		Y	3	4	TM
205	809_00350	Cytochrome c biogenesis protein CcsA	Core			8	8	TM
206	809_00353	hypothetical protein	Core			2	2	TM
207	809_00354	1,4-dihydroxy-2-naphthoate octaprenyltransferase	Core			8	6	TM
208	809_00358	Putative cryptic C4-dicarboxylate transporter DcuD	Core		Y	10	11	TM
209	809_00371	preprotein translocase subunit SecE	Core			1	1	TM
210	809_00384	Manganese transport system membrane protein MntB	Core			9	8	TM
211	809_00391	Sensor histidine kinase DesK	Core			7	7	TM
212	809_00393	hypothetical protein	Core			9	9	TM
213	809_00394	hypothetical protein	Core			1	1	TM
214	809_00395	hypothetical protein	Core			9	9	TM
215	809_00397	ABC-2 family transporter protein	Core			6	6	TM
216	809_00398	hypothetical protein	Core			3	3	TM
217	809_00399	putative permease	Core			9	8	TM
218	809_00409	hypothetical protein	Core		Y	1	2	TM
219	809_00412	hypothetical protein	Core			1	1	TM
220	809_00428	Oligopeptide transport system permease protein OppC	Core			6	6	TM
221	809_00429	Oligopeptide transport system permease protein OppB	Core		Y	5	4	TM
222	809_00433	Serine transporter	Core			11	11	TM
223	809_00446	Maltose transport system permease protein MalF	Core			8	8	TM
224	809_00447	Trehalose transport system permease protein SugB	Core			6	6	TM
225	809_00448	TraX protein	Core			8	8	TM
226	809_00449	Nitrite transporter NirC	Core			6	6	TM
227	809_00452	preprotein translocase subunit SecY	Core		Y	9	10	TM
228	809_00466	Putative transmembrane protein (PGPGW)	Core			3	3	TM
229	809_00482	Aspartate/alanine antiporter	Core			11	11	TM
230	809_00495	hypothetical protein	Core			11	11	TM
231	809_00498	PspC domain protein	Core			4	4	TM
232	809_00499	nitrate/nitrite sensor protein NarQ	Core			5	5	TM
233	809_00503	Methionine import system permease protein MetP	Core			5	5	TM
234	809_00507	Bacterial membrane flanked domain protein	Core			2	2	TM
235	809_00511	Manganese transport system membrane protein MntB	Core			7	7	TM
236	809_00512	Manganese transport system membrane protein MntB	Core			9	8	TM
237	809_00522	Hemin transport system permease protein HmuU	Core		Y	8	8	TM
238	809_00527	Inner membrane transport protein YdhP	Core			12	12	TM
239	809_00530	hypothetical protein	Core		Y	1	2	TM
240	809_00534	Inner membrane protein YhjD	Core			7	7	TM
241	809_00546	putative multidrug resistance protein EmrY	Core			14	14	TM
242	809_00551	O-acetyltransferase OatA	Core			11	11	TM
243	809_00560	hypothetical protein	Core			2	2	TM
244	809_00570	hypothetical protein	Core			2	2	TM
245	809_00571	Trehalose transport system permease protein SugB	Core			6	6	TM
246	809_00572	Trehalose transport system permease protein SugA	Core			6	5	TM
247	809_00575	Transcriptional regulator LytR	Core			1	1	TM
248	809_00584	hypothetical protein	Core			1	1	TM
249	809_00590	Sensor histidine kinase MtrB	Core		Y	1	1	TM
250	809_00607	hypothetical protein	Core			4	4	TM
251	809_00609	hypothetical protein	Core	Y		1	1	TM
252	809_00612	hypothetical protein	Core			1	1	TM
253	809_00625	hypothetical protein	Core			7	7	TM
254	809_00631	hypothetical protein	Core			10	10	TM
255	809_00636	D-serine/D-alanine/glycine transporter	Core			12	12	TM
256	809_00646	p-aminobenzoyl-glutamate transport protein	Core			12	12	TM
257	809_00648	Cell division protein FtsX	Core			4	4	TM
258	809_00654	Iron-uptake system permease protein FeuB	Core			9	8	TM
259	809_00655	Iron-uptake system permease protein FeuC	Core			10	10	TM
260	809_00697	Glutathione transport system permease protein GsiC	Core			6	5	TM
261	809_00712	Inner membrane protein YccF	Core			3	3	TM
262	809_00724	putative glutamine ABC transporter permease protein GlnM	Core			3	5	TM
263	809_00737	Signal transduction histidine-protein kinase/phosphatase MprB	Core			2	2	TM
264	809_00748	Putative oxidoreductase MhqP	Core		Y	2	3	TM
265	809_00749	hypothetical protein	Core			4	3	TM

266	809_00750	putative dolichyl-phosphate-mannose--protein mannosyltransferase	Core		11	10	TM
267	809_00751	Glycine betaine transporter BetP	Core		12	12	TM
268	809_00752	hypothetical protein	Core	Y	9	10	TM
269	809_00763	hypothetical protein	Core		1	1	TM
270	809_00765	Nicotinamide mononucleotide transporter	Core		7	5	TM
271	809_00772	hypothetical protein	Core		6	6	TM
272	809_00789	Arginine/ornithine antiporter	Core		13	14	TM
273	809_00793	Cell division protein FtsL	Core		1	1	TM
274	809_00799	Bax inhibitor 1 like protein	Core		7	7	TM
275	809_00804	hypothetical protein	Core		1	1	TM
276	809_00809	Multidrug resistance protein 3	Core		14	14	TM
277	809_00815	Multidrug resistance protein 3	Core		14	14	TM
278	809_00819	hypothetical protein	Core		1	1	TM
279	809_00823	hypothetical protein	Core		3	3	TM
280	809_00824	pheromone autoinducer 2 transporter	Core		8	8	TM
281	809_00833	Putative HMP/thiamine permease protein YkoE	Core		6	5	TM
282	809_00836	Energy-coupling factor transporter transmembrane protein EcfT	Core		6	4	TM
283	809_00840	Dipeptide transport system permease protein DppB	Core		6	6	TM
284	809_00853	hypothetical protein	Core		4	4	TM
285	809_00856	GtrA-like protein	Core		4	4	TM
286	809_00858	GABA permease	Core		12	12	TM
287	809_00860	GABA permease	Core		12	12	TM
288	809_00878	hypothetical protein	Core		2	2	TM
289	809_00890	Sodium/glucose cotransporter	Core		14	14	TM
290	809_00891	hypothetical protein	Core		2	2	TM
291	809_00897	hypothetical protein	Core		4	4	TM
292	809_00899	Sodium/proline symporter	Core		13	12	TM
293	809_00906	PspC domain protein	Core		1	1	TM
294	809_00926	Decaprenyl-phosphate N-acetylglucosaminephosphotransferase	Core		11	11	TM
295	809_00928	ATP synthase subunit a	Core		6	6	TM
296	809_00929	ATP synthase subunit c	Core		2	2	TM
297	809_00930	ATP synthase subunit b	Core		1	1	TM
298	809_00936	hypothetical protein	Core		1	1	TM
299	809_00958	putative multidrug resistance protein EmrY	Core		14	14	TM
300	809_00960	Hemin transport system permease protein HmuU	Core		10	9	TM
301	809_00968	Arginine exporter protein ArgO	Core		6	5	TM
302	809_00974	Low molecular weight protein antigen 6	Core		2	1	TM
303	809_00980	Cadmium, cobalt and zinc/H(+)-K(+) antiporter	Core		6	6	TM
304	809_00987	hypothetical protein	Core		2	2	TM
305	809_01000	Dipeptide transport system permease protein DppB	Core		6	6	TM
306	809_01001	Glutathione transport system permease protein GsiD	Core		6	6	TM
307	809_01013	hypothetical protein	Core		1	1	TM
308	809_01021	Sulfite exporter TauE/SafE	Core	Y	6	8	TM
309	809_01024	Inner membrane amino-acid ABC transporter permease protein YecS	Core		6	4	TM
310	809_01026	hypothetical protein	Core		8	8	TM
311	809_01032	PTS system beta-glucoside-specific EIIBCA component	Core		10	11	TM
312	809_01045	hypothetical protein	Core		2	2	TM
313	809_01069	hypothetical protein	Core		1	1	TM
314	809_01076	Di-/tripeptide transporter	Core		14	14	TM
315	809_01083	Anaerobic C4-dicarboxylate transporter DcuB	Core		11	11	TM
316	809_01085	Putative esterase	Core		4	4	TM
317	809_01086	Lysylphosphatidylglycerol biosynthesis bifunctional protein LysX	Core		12	12	TM
318	809_01088	putative siderophore transport system permease protein YfiZ precursor	Core		9	9	TM
319	809_01089	Fe(3+) dicitrate transport system permease protein FecD	Core		9	9	TM
320	809_01092	Major Facilitator Superfamily protein	Core	Y	11	11	TM
321	809_01105	Magnesium and cobalt efflux protein CorC	Core	Y	3	4	TM
322	809_01106	Magnesium and cobalt efflux protein CorC	Core		3	3	TM
323	809_01110	Magnesium transport protein CorA	Core		2	2	TM
324	809_01115	NADH dehydrogenase-like protein	Core		1	1	TM
325	809_01117	Inner membrane metabolite transport protein YhjE	Core		12	12	TM
326	809_01119	Sodium Bile acid symporter family protein	Core		9	9	TM
327	809_01126	Apolipoprotein N-acyltransferase	Core		8	7	TM
328	809_01139	Sec-independent protein translocase protein TatC	Core		6	6	TM
329	809_01153	Anaerobic C4-dicarboxylate transporter DcuA	Core		11	10	TM
330	809_01160	Undecaprenyl-diphosphatase	Core		6	5	TM
331	809_01164	hypothetical protein	Core		2	2	TM
332	809_01165	hypothetical protein	Core		4	4	TM

333	809_01177	TVP38/TMEM64 family inner membrane protein YdjZ	Core		6	6	TM
334	809_01180	hypothetical protein	Core	Y	1	2	TM
335	809_01326	preprotein translocase subunit SecD	Core		6	6	TM
336	809_01340	CDP-diacylglycerol--inositol 3-phosphatidyltransferase	Core		3	4	TM
337	809_01355	Alpha-(1->3)-arabinofuranosyltransferase	Core		12	10	TM
338	809_01362	hypothetical protein	Core		6	5	TM
339	809_01365	hypothetical protein	Core		2	2	TM
340	809_01368	Polyphosphate glucokinase	Core			1	TM
341	809_01373	hypothetical protein	Core		2	2	TM
342	809_01385	hypothetical protein	Core		5	5	TM
343	809_01395	PTS system fructose-specific EIIABC component	Core		8	9	TM
344	809_01400	Putative pyrimidine permease RutG	Core		12	11	TM
345	809_01403	hypothetical protein	Core		1	1	TM
346	809_01409	hypothetical protein	Core		4	4	TM
347	809_01413	Biotin transporter BioY	Core		6	6	TM
348	809_01415	Energy-coupling factor transporter transmembrane protein EcfT	Core		5	4	TM
349	809_01419	Putative CDP-diacylglycerol--glycerol-3-phosphate 3-phosphatidyl-transferase 2	Core		4	4	TM
350	809_01421	Inner membrane protein alx	Core		9	9	TM
351	809_01422	DNA translocase SpoIIIE	Core		4	5	TM
352	809_01435	DNA-damage-inducible protein F	Core		12	12	TM
353	809_01461	Zinc metalloprotease Rip1	Core		4	4	TM
354	809_01464	hypothetical protein	Core		2	2	TM
355	809_01466	hypothetical protein	Core		2	2	TM
356	809_01467	Phosphatidate cytidylyltransferase	Core		8	7	TM
357	809_01486	Signal peptidase I	Core		1	1	TM
358	809_01498	Signal recognition particle receptor FtsY	Core		1	1	TM
359	809_01502	Amino-acid carrier protein AlsT	Core		9	9	TM
360	809_01515	Electron transfer DM13	Core		1	1	TM
361	809_01517	Prolipoprotein diacylglyceryl transferase	Core		7	7	TM
362	809_01519	Tryptophan-associated transmembrane protein (Trp_oprn_chp)	Core	Y	3	4	TM
363	809_01525	putative transporter	Core		12	12	TM
364	809_01526	hypothetical protein	Core		2	2	TM
365	809_01531	hypothetical protein	Core		4	4	TM
366	809_01540	hypothetical protein	Core		2	2	TM
367	809_01544	EamA-like transporter family protein	Core	Y	9	9	TM
368	809_01547	Lipoprotein signal peptidase	Core		3	4	TM
369	809_01555	YGGT family protein	Core		3	2	TM
370	809_01564	Phospho-N-acetylmuramoyl-pentapeptide-transferase	Core		10	10	TM
371	809_01567	Penicillin-binding protein PbpB	Core		1	1	TM
372	809_01568	Cell division protein FtsL	Core		1	1	TM
373	809_01571	hypothetical protein	Core		2	2	TM
374	809_01581	Acyltransferase family protein	Core		8	7	TM
375	809_01587	Menaquinol-cytochrome c reductase cytochrome b subunit	Core		9	9	TM
376	809_01589	Menaquinol-cytochrome c reductase cytochrome c subunit	Core		2	2	TM
377	809_01590	Cytochrome c oxidase subunit 3	Core		5	5	TM
378	809_01592	Cytochrome c oxidase polypeptide 4	Core		3	3	TM
379	809_01596	hypothetical protein	Core		3	2	TM
380	809_01611	putative siderophore transport system permease protein YfhA	Core		8	8	TM
381	809_01615	hypothetical protein	Core		2	2	TM
382	809_01616	RDD family protein	Core		2	3	TM
383	809_01630	putative sulfoacetate transporter SauU	Core		12	11	TM
384	809_01646	SURF1 family protein	Core		2	2	TM
385	809_01647	cobalamin biosynthesis protein	Core		2	1	TM
386	809_01656	hypothetical protein	Core		5	7	TM
387	809_01663	Guanyl-specific ribonuclease Sa	Core		1	1	TM
388	809_01666	hypothetical protein	Core		1	1	TM
389	809_01669	hypothetical protein	Core		1	1	TM
390	809_01673	VIT family protein	Core		5	4	TM
391	809_01688	hypothetical protein	Core		1	1	TM
392	809_01695	Putative multidrug export ATP-binding/permease protein	Core		6	5	TM
393	809_01696	Branched-chain amino acid transport system 2 carrier protein	Core		12	12	TM
394	809_01699	Glycine betaine transporter OpuD	Core		12	12	TM
395	809_01700	hypothetical protein	Core		2	2	TM
396	809_01701	Membrane transport protein	Core	Y	7	8	TM
397	809_01704	Glutathione transport system permease protein GsiD	Core		6	6	TM
398	809_01709	putative inner membrane protein	Core		9	8	TM
399	809_01712	Glutathione transport system permease protein GsiD	Core		12	12	TM

400	809_01714	Multidrug resistance protein MdtH	Core		11	11	TM
401	809_01721	Threonine efflux protein	Core		6	6	TM
402	809_01725	ComE operon protein 1	Core		1	1	TM
403	809_01733	Aspartate/alanine antiporter	Core		11	10	TM
404	809_01744	hypothetical protein	Core		3	3	TM
405	809_01762	putative MscS family protein YkuT	Core		3	3	TM
406	809_01764	hypothetical protein	Core	Y	8	10	TM
407	809_01774	hypothetical protein	Core		6	5	TM
408	809_01777	ABC-2 family transporter protein	Core		6	6	TM
409	809_01779	Cytochrome c oxidase caa3 assembly factor (Caa3_CtaG)	Core		16	16	TM
410	809_01780	Carbon starvation protein A	Core		16	15	TM
411	809_01794	hypothetical protein	Core		1	1	TM
412	809_01800	Sensor histidine kinase DesK	Core	Y	5	5	TM
413	809_01802	hypothetical protein	Core		8	8	TM
414	809_01804	hypothetical protein	Core		2	2	TM
415	809_01805	hypothetical protein	Core		3	3	TM
416	809_01810	Rhomboid family protein	Core		6	6	TM
417	809_01835	Copper-exporting P-type ATPase A	Core		6	6	TM
418	809_01838	Methylamine utilisation protein MauE	Core		3	3	TM
419	809_01842	ABC transporter permease YtrF precursor	Core		10	10	TM
420	809_01860	Cytochrome bd-I ubiquinol oxidase subunit 2	Core		9	8	TM
421	809_01862	putative ABC transporter ATP-binding protein	Core		6	5	TM
422	809_01868	Phosphate transport system permease protein PstA	Core		6	6	TM
423	809_01883	Haemolysin-III related	Core		7	7	TM
424	809_01893	Ktr system potassium uptake protein B	Core		9	9	TM
425	809_01897	putative sensor histidine kinase TcrY	Core		2	2	TM
426	809_01899	Antiseptic resistance protein	Core		13	13	TM
427	809_01902	Uric acid transporter UacT	Core		13	11	TM
428	809_01905	hypothetical protein	Core		10	8	TM
429	809_01908	hypothetical protein	Core		8	8	TM
430	809_01923	hypothetical protein	Core		1	1	TM
431	809_01926	Inner membrane protein YjjP	Core		11	10	TM
432	809_01929	hypothetical protein	Core	Y	5	6	TM
433	809_01934	hypothetical protein	Core		2	2	TM
434	809_01937	SPFH domain / Band 7 family protein	Core		2	2	TM
435	809_01939	Proton glutamate symport protein	Core		8	8	TM
436	809_01941	High-affinity choline transport protein	Core		12	12	TM
437	809_01945	hypothetical protein	Core		2	2	TM
438	809_01946	hypothetical protein	Core		4	4	TM
439	809_01951	ATP-dependent zinc metalloprotease FtsH	Core		2	2	TM
440	809_01957	Aerobic C4-dicarboxylate transport protein	Core		6	6	TM
441	809_01974	Na(+)/H(+) antiporter subunit A	Core		25	25	TM
442	809_01975	Na(+)/H(+) antiporter subunit C	Core		3	3	TM
443	809_01976	Na(+)/H(+) antiporter subunit D	Core		13	13	TM
444	809_01977	putative monovalent cation/H+ antiporter subunit E	Core		2	2	TM
445	809_01978	putative monovalent cation/H+ antiporter subunit F	Core		3	3	TM
446	809_01979	Na(+)/H(+) antiporter subunit G	Core		3	3	TM
447	809_01980	Polyprenol-phosphate-mannose-dependent alpha-(1-2)-phosphatidylinositol pentamannoside mannosyltransferase	Core		11	9	TM
448	809_01982	hypothetical protein	Core		1	1	TM
449	809_01987	putative cardiolipin synthase YwiE	Core		2	2	TM
450	809_01988	ABC-2 family transporter protein	Core		6	6	TM
451	809_01991	Four helix bundle sensory module for signal transduction	Core		3	4	TM
452	809_02001	Disulfide bond formation protein DsbB	Core		5	5	TM
453	809_02016	hypothetical protein	Core		6	3	TM
454	809_02040	hypothetical protein	Core		12	12	TM
455	809_02043	Sodium/glutamate symporter	Core		13	13	TM
456	809_02052	Putative efflux system component YknX	Core		1	1	TM
457	809_02066	Sortase family protein	Core		2	2	TM
458	809_02069	Oligopeptide transport ATP-binding protein OppD	Core		5	5	TM
459	809_02070	Nickel transport system permease protein NikB	Core		7	6	TM
460	809_02072	Sulfate permease CysP	Core		12	12	TM
461	809_02074	Filamentous hemagglutinin	Core		6	6	TM
462	809_02075	hypothetical protein	Core		2	1	TM
463	809_02078	Hemin transport system permease protein HmuU	Core		10	10	TM
464	809_02087	EamA-like transporter family protein	Core		10	10	TM
465	809_02090	Lysylphosphatidylglycerol biosynthesis bifunctional protein LysX	Core		6	7	TM
466	809_02107	hypothetical protein	Core		1	1	TM

467	809_02109	O-acetyltransferase OatA	Core						9	9		TM
468	809_02110	hypothetical protein	Core						2	2		TM
469	809_02111	hypothetical protein	Core						12	9		TM
470	809_02117	Membrane protein YdfJ	Core						11	11		TM
471	809_02118	hypothetical protein	Core		Y				7	8		TM
472	809_02119	hypothetical protein	Core		Y				3	4		TM
473	809_02128	hypothetical protein	Core		Y				8	9		TM
474	809_02130	hypothetical protein	Core						11	11		TM
475	809_02137	Glycerol-3-phosphate transporter	Core						12	12		TM
476	809_02153	CAAX amino terminal protease self- immunity	Core						7	6		TM
477	809_02160	Nucleoside permease NupX	Core		Y				8	8		TM
478	809_02168	Inner membrane transport protein YnfM	Core						12	12		TM
479	809_02176	Sensor histidine kinase LiaS	Core						5	5		TM
480	809_02178	hypothetical protein	Core						1	1		TM
481	809_02179	Sortase family protein	Core						1	1		TM
482	809_02180	Membrane protein insertase MisCA precursor	Core						6	6		TM
483	809_02182	hypothetical protein	Core						6	6		TM
484	809_02183	hypothetical protein	Core						3	3		TM
485	809_01087	Fe(3+)-citrate-binding protein YfmC precursor	Core	Y		SpII	Lipoprotein		1	1		TM-Lipo
486	809_01593	Cytochrome c oxidase subunit 2 precursor	Core		Y		Lipoprotein	Lipobox	2	3		TM-Lipo
487	809_00041	Ferric enterobactin transport system permease protein FepG	Core	Y	Y				8	9		TM-Sec
488	809_00253	hypothetical protein	Core	Y	Y				5	6		TM-Sec
489	809_00455	Sialidase precursor	Core	Y	Y				1	1		TM-Sec
490	809_00520	Htaa	Core	Y	Y				1	2		TM-Sec
491	809_00535	D-alanyl-D-alanine carboxypeptidase DacB precursor	Core	Y	Y				1	1		TM-Sec
492	809_00698	Oligopeptide transport system permease protein OppC	Core	Y	Y				5	6		TM-Sec
493	809_01678	Magnesium and cobalt efflux protein CorC	Core	Y	Y				2	3		TM-Sec
494	809_01837	hypothetical protein	Core	Y					1	1		TM-Sec
495	809_02044	hypothetical protein	Core	Y	Y				1	1		TM-Sec
496	809_02134	hypothetical protein	Core	Y	Y				1	2		TM-Sec
497	809_02156	Htaa	Core	Y	Y				1	1		TM-Sec
498	809_02064	hypothetical protein	Core	Y	Y				1	2	sortaseA	LPXTG
499	809_02065	hypothetical protein	Core	Y	Y				1	2	sortaseD	LPXTG
500	809_00055	Sensor protein VraS	Accessory		Y				3			Amb
501	809_00088	hypothetical protein	Accessory						1			Amb
502	809_00217	hypothetical protein	Accessory									Amb
503	809_00266	hypothetical protein	Accessory		Y					1		Amb
504	809_00295	dTDP-glucose 4,6-dehydratase	Accessory		Y							Amb
505	809_00352	hypothetical protein	Accessory		Y					1		Amb
506	809_00484	tRNA threonylcarbamoyladenosine biosynthesis protein TsaB	Accessory		Y							Amb
507	809_00531	hypothetical protein	Accessory						1			Amb
508	809_00633	hypothetical protein	Accessory									Amb
509	809_00669	Glutamine cyclotransferase	Accessory		Y							Amb
510	809_00686	hypothetical protein	Accessory		Y							Amb
511	809_00717	Murein DD-endopeptidase MepM	Accessory	Y						1		Amb
512	809_00738	Periplasmic pH-dependent serine endoprotease DegQ precursor	Accessory						1			Amb
513	809_00742	hypothetical protein	Accessory		Y					1		Amb
514	809_00777	Nitronate monooxygenase	Accessory		Y							Amb
515	809_01097	hypothetical protein	Accessory							1		Amb
516	809_01167	PTS-dependent dihydroxyacetone kinase, phosphotransferase subunit DhaM	Accessory		Y							Amb
517	809_01194	hypothetical protein	Accessory		Y					1		Amb
518	809_01198	hypothetical protein	Accessory		Y							Amb
519	809_01204	hypothetical protein	Accessory						1			Amb
520	809_01304	putative aminodeoxychorismate lyase	Accessory		Y					1		Amb
521	809_01308	Phosphotransferase enzyme family protein	Accessory						1			Amb
522	809_01327	preprotein translocase subunit YajC	Accessory		Y					1		Amb
523	809_01332	hypothetical protein	Accessory						1			Amb
524	809_01462	1-deoxy-D-xylulose 5-phosphate reductoisomerase	Accessory		Y							Amb
525	809_01472	Peptidase family M23	Accessory		Y					1		Amb
526	809_01475	Competence protein ComM	Accessory		Y							Amb
527	809_01478	Thiamine-phosphate synthase	Accessory						1			Amb
528	809_01479	Hydrogen cyanide synthase subunit HcnC precursor	Accessory		Y							Amb
529	809_01545	hypothetical protein	Accessory		Y					1		Amb
530	809_01717	hypothetical protein	Accessory		Y							Amb
531	809_01740	hypothetical protein	Accessory		Y					1		Amb
532	809_01776	YcaO-like family protein	Accessory		Y							Amb
533	809_02004	hypothetical protein	Accessory		Y					1		Amb

534	809_02008	Thiol-disulfide oxidoreductase ResA	Accessory	Y	Y			1	Amb
535	809_02055	hypothetical protein	Accessory		Y				Amb
536	809_02138	UDP-galactopyranose mutase	Accessory		Y				Amb
537	809_02257	hypothetical protein	Accessory		Y				Amb
538	809_02260	zeta-carotene-forming phytoene desaturase	Accessory		Y				Amb
539	BRAD22_01704	hypothetical protein	Accessory		Y			1	Amb
540	BRAD22_01735	hypothetical protein	Accessory		Y			1	Amb
541	BRAD22_01737	hypothetical protein	Accessory				2		Amb
542	BRAD22_01741	Phage-related minor tail protein	Accessory					2	Amb
543	BRAD22_01794	hypothetical protein	Accessory				1		Amb
544	BRAD22_01874	Phage portal protein	Accessory		Y				Amb
545	BRAD22_01887	Tyrosine recombinase XerC	Accessory						Amb
546	BRAD22_02013	hypothetical protein	Accessory		Y			1	Amb
547	BRAD22_02028	hypothetical protein	Accessory		Y				Amb
548	BRAD22_02042	hypothetical protein	Accessory				1		Amb
549	BRAD22_02343	hypothetical protein	Accessory		Y				Amb
550	BRAD22_02416	hypothetical protein	Accessory		Y				Amb
551	0102_00100	hypothetical protein	Accessory		Y				Amb
552	0102_00181	Helix-turn-helix	Accessory		Y				Amb
553	0102_00184	hypothetical protein	Accessory		Y			1	Amb
554	0102_00559	Phage-related minor tail protein	Accessory					2	Amb
555	0102_01297	hypothetical protein	Accessory		Y			1	Amb
556	0102_01429	preprotein translocase subunit YajC	Accessory		Y			1	Amb
557	0102_01795	hypothetical protein	Accessory				1		Amb
558	0102_02186	hypothetical protein	Accessory		Y				Amb
559	2590_00023	Sec-independent protein translocase protein TatAy	Accessory		Y			1	Amb
560	2590_00981	Epidermin decarboxylase	Accessory		Y				Amb
561	2590_01065	Murein DD-endopeptidase MepM	Accessory		Y			1	Amb
562	2590_01871	hypothetical protein	Accessory	Y					Amb
563	BRAD-2649_00099	hypothetical protein	Accessory		Y				Amb
564	BRAD-2649_00785	Signal-transduction histidine kinase senX3	Accessory		Y			1	Amb
565	BRAD-2649_00975	hypothetical protein	Accessory		Y				Amb
566	BRAD-2649_00977	Transposase IS116/IS110/IS902 family protein	Accessory		Y				Amb
567	BRAD-2649_01068	Aerobic C4-dicarboxylate transport protein	Accessory						Amb
568	BRAD-2649_01814	hypothetical protein	Accessory		Y			1	Amb
569	BRAD-2649_01815	hypothetical protein	Accessory		Y				Amb
570	BRAD-2649_02079	hypothetical protein	Accessory		Y				Amb
571	4940_00155	hypothetical protein	Accessory						Amb
572	4940_01941	Helix-turn-helix domain protein	Accessory		Y				Amb
573	131001_00055	hypothetical protein	Accessory		Y				Amb
574	131001_00889	hypothetical protein	Accessory		Y				Amb
575	131001_00897	hypothetical protein	Accessory		Y				Amb
576	131002_00157	hypothetical protein	Accessory		Y			1	Amb
577	131002_00252	hypothetical protein	Accessory		Y			1	Amb
578	131002_00336	hypothetical protein	Accessory		Y			1	Amb
579	131002_00522	putative endopeptidase precursor	Accessory		Y				Amb
580	131002_00639	hypothetical protein	Accessory				2		Amb
581	131002_00648	Glutamine cyclotransferase	Accessory		Y	SpII			Amb
582	131002_00749	Nitronate monooxygenase	Accessory		Y				Amb
583	131002_00926	hypothetical protein	Accessory					1	Amb
584	131002_01144	PTS-dependent dihydroxyacetone kinase, phosphotransferase subunit DhaM	Accessory		Y				Amb
585	131002_01234	hypothetical protein	Accessory				1		Amb
586	131002_01402	Competence protein ComM	Accessory		Y				Amb
587	131002_01405	Thiamine-phosphate synthase	Accessory				1		Amb
588	131002_01406	Hydrogen cyanide synthase subunit HcnC precursor	Accessory		Y				Amb
589	131002_01472	hypothetical protein	Accessory		Y			1	Amb
590	131002_01623	hypothetical protein	Accessory		Y			1	Amb
591	131002_01759	hypothetical protein	Accessory		Y		1		Amb
592	131002_01772	hypothetical protein	Accessory		Y				Amb
593	131002_01937	hypothetical protein	Accessory		Y			1	Amb
594	131002_01990	hypothetical protein	Accessory		Y				Amb
595	210931_01616	Helix-turn-helix domain protein	Accessory		Y				Amb
596	210931_02099	hypothetical protein	Accessory		Y				Amb
597	04-3911_02223	hypothetical protein	Accessory		Y			2	Amb
598	04-7514_00218	hypothetical protein	Accessory		Y			1	Amb
599	04-7514_00640	hypothetical protein	Accessory				1		Amb
600	04-7514_00657	putative endopeptidase precursor	Accessory		Y				Amb

601	04-7514_00676	putative propionyl-CoA carboxylase beta chain 5	Accessory							1		Amb
602	04-7514_00859	hypothetical protein	Accessory		Y					1		Amb
603	04-7514_01040	hypothetical protein	Accessory		Y							Amb
604	04-7514_01248	hypothetical protein	Accessory		Y							Amb
605	04-7514_01547	1-deoxy-D-xylulose 5-phosphate reductoisomerase	Accessory		Y							Amb
606	04-7514_02371	15-cis-phytoene synthase	Accessory						1			Amb
607	210932_01997	hypothetical protein	Accessory						1			Amb
608	FRC11_01252	hypothetical protein	Accessory		Y							Amb
609	FRC58_00303	hypothetical protein	Accessory		Y					1		Amb
610	FRC58_01683	YcfA-like protein	Accessory						1			Amb
611	KZN-2016-48390_00258	RNA polymerase sigma factor SigA	Accessory									Amb
612	KZN-2016-48390_00260	hypothetical protein	Accessory	Y								Amb
613	LSPQ-04227_00246	UDP-N-acetylmuramoyl-L-alanyl-D-glutamate synthetase	Accessory									Amb
614	LSPQ-04227_00427	hypothetical protein	Accessory		Y							Amb
615	LSPQ-04227_00577	hypothetical protein	Accessory		Y							Amb
616	LSPQ-04227_00676	ATP-dependent DNA helicase UvrD2	Accessory									Amb
617	LSPQ-04227_01176	hypothetical protein	Accessory		Y							Amb
618	LSPQ-04227_01652	hypothetical protein	Accessory	Y								Amb
619	LSPQ-04227_01739	YceG-like family protein	Accessory		Y					1		Amb
620	LSPQ-04227_01821	hypothetical protein	Accessory		Y					1		Amb
621	LSPQ-04227_01939	Copper transporter MctB precursor	Accessory		Y					1		Amb
622	LSPQ-04227_02153	hypothetical protein	Accessory		Y							Amb
623	LSPQ-04227_02328	Dehydrosqualene desaturase	Accessory		Y							Amb
624	LSPQ-04228_01067	hypothetical protein	Accessory		Y					1		Amb
625	LSPQ-04228_01338	Helix-turn-helix domain protein	Accessory		Y							Amb
626	LSPQ-04228_01354	hypothetical protein	Accessory		Y							Amb
627	LSPQ-04228_01356	hypothetical protein	Accessory							1		Amb
628	LSPQ-04228_01950	hypothetical protein	Accessory		Y					1		Amb
629	NCTC12077_01414	hypothetical protein	Accessory							2		Amb
630	NCTC12077_02412	enterobactin synthase subunit F	Accessory		Y							Amb
631	BRAD-2649_01020	hypothetical protein	Accessory		Y					1		amb
632	809_00634	hypothetical protein	Accessory						2	2	sortaseD	Amb
633	BRAD22_01929	hypothetical protein	Accessory						1	1	sortaseD	Amb
634	4940_01627	Fimbrial subunit type 1 precursor	Accessory						1	1	sortaseA	Amb
635	4940_02225	hypothetical protein	Accessory						1	1	sortaseA	Amb
636	131001_01812	hypothetical protein	Accessory						1	1	sortaseD	Amb
637	210931_01789	hypothetical protein	Accessory						1	1	sortaseD	Amb
638	KZN-2016-48390_01941	Fimbrial subunit type 1 precursor	Accessory						1	1	sortaseA	Amb
639	LSPQ-04227_02156	Fimbrial subunit type 1 precursor	Accessory						1	1	sortaseA	Amb
640	NCTC12077_02488	hypothetical protein	Accessory						1	1	sortaseA	Amb
641	LSPQ-04227_00420	Manganese ABC transporter substrate-binding lipoprotein precursor	Accessory		Y		Lipoprotein	Lipobox				Sec-Lipo
642	809_00016	TMAO reductase system periplasmic protein TorT	Accessory	Y	Y	SpII	Lipoprotein					Sec-Lipo
643	809_00043	putative siderophore-binding lipoprotein YfiY precursor	Accessory	Y	Y	SpII	Lipoprotein			1		Sec-Lipo
644	809_00062	corrinoid ABC transporter substrate-binding protein	Accessory	Y	Y	SpII	Lipoprotein					Sec-Lipo
645	809_00348	Vitamin B12-binding protein precursor	Accessory	Y	Y	SpII	Lipoprotein					Sec-Lipo
646	809_00567	corrinoid ABC transporter substrate-binding protein	Accessory	Y	Y	SpII	Lipoprotein					Sec-Lipo
647	809_00679	Superoxide dismutase [Cu-Zn] precursor	Accessory	Y	Y	SpII	Lipoprotein					Sec-Lipo
648	809_00723	ABC transporter glutamine-binding protein GlnH precursor	Accessory	Y	Y	SpII	Lipoprotein					Sec-Lipo
649	809_01162	hypothetical protein	Accessory			SpII	Lipoprotein					Sec-Lipo
650	809_01324	Bacterial extracellular solute-binding proteins, family 5 Middle	Accessory	Y	Y	SpII	Lipoprotein					Sec-Lipo
651	809_01532	hypothetical protein	Accessory	Y	Y	SpII	Lipoprotein					Sec-Lipo
652	809_01713	Periplasmic dipeptide transport protein precursor	Accessory	Y	Y	SpII	Lipoprotein					Sec-Lipo
653	809_02268	3-phenylpropionate/cinnamic acid dioxygenase ferredoxin subunit	Accessory		Y	SpII	Lipoprotein					Sec-Lipo
654	BRAD22_01723	Periplasmic dipeptide transport protein precursor	Accessory	Y	Y	SpII	Lipoprotein					Sec-Lipo
655	BRAD22_01763	hypothetical protein	Accessory	Y	Y	SpII	Lipoprotein					Sec-Lipo
656	BRAD22_02326	hypothetical protein	Accessory		Y	SpII	Lipoprotein	Lipobox		1		Sec-Lipo
657	0102_00541	hypothetical protein	Accessory	Y	Y	SpII	Lipoprotein					Sec-Lipo
658	2590_01059	hypothetical protein	Accessory	Y	Y	SpII	Lipoprotein					Sec-Lipo
659	2590_01060	hypothetical protein	Accessory	Y	Y	SpII	Lipoprotein					Sec-Lipo
660	2590_01066	hypothetical protein	Accessory	Y	Y	SpII	Lipoprotein					Sec-Lipo
661	2590_01483	corrinoid ABC transporter substrate-binding protein	Accessory	Y	Y	SpII	Lipoprotein					Sec-Lipo
662	BRAD-2649_00980	Carboxylesterase A precursor	Accessory	Y	Y	SpII	Lipoprotein					Sec-Lipo
663	04-7514_01291	hypothetical protein	Accessory		Y	SpII	Lipoprotein					Sec-Lipo
664	LSPQ-04227_00070	putative siderophore-binding lipoprotein YfiY precursor	Accessory	Y	Y	SpII	Lipoprotein			1		Sec-Lipo
665	LSPQ-04227_01622	ABC transporter glutamine-binding protein GlnH precursor	Accessory	Y	Y	SpII	Lipoprotein					Sec-Lipo
666	LSPQ-04228_00360	Lipoprotein LpqB precursor	Accessory	Y	Y	SpII	Lipoprotein					Sec-Lipo
667	0102_00104	hypothetical protein	Accessory	Y	Y		Lipoprotein	Lipobox				Sec-Lipo

668	KZN-2016-48390_02092	hypothetical protein	Accessory	Y	Y		Lipoprotein	Lipobox	1	Sec-Lipo
669	131002_00171	hypothetical protein	Accessory	Y	Y		Lipoprotein	No		Sec-Lipo
670	LSPQ-04227_00715	hypothetical protein	Accessory	Y	Y	SpII	Lipoprotein			Sec-Lipo
671	809_02237	Periplasmic dipeptide transport protein precursor	Accessory		Y		Lipoprotein	Lipobox		Sec-Lipo
672	809_00014	hypothetical protein	Accessory	Y	Y				1	Sec-SpI
673	809_00091	hypothetical protein	Accessory	Y	Y				1	Sec-SpI
674	809_00102	Alpha-amylase precursor	Accessory	Y	Y					Sec-SpI
675	809_00185	Ribosome inactivating protein	Accessory	Y	Y				1	Sec-SpI
676	809_00186	hypothetical protein	Accessory	Y	Y				1	Sec-SpI
677	809_00189	hypothetical protein	Accessory	Y	Y					Sec-SpI
678	809_00216	hypothetical protein	Accessory	Y	Y					Sec-SpI
679	809_00240	LGFP repeat protein	Accessory	Y	Y				1	Sec-SpI
680	809_00241	hypothetical protein	Accessory	Y	Y				1	Sec-SpI
681	809_00242	Short repeats of unknown function	Accessory	Y	Y					Sec-SpI
682	809_00298	Diacylglycerol acyltransferase/mycolyltransferase Ag85A precursor	Accessory	Y	Y				1	Sec-SpI
683	809_00444	hypothetical protein	Accessory	Y	Y				1	Sec-SpI
684	809_00538	putative endopeptidase precursor	Accessory	Y	Y					Sec-SpI
685	809_00635	hypothetical protein	Accessory	Y	Y					Sec-SpI
686	809_00666	Resuscitation-promoting factor RpfA precursor	Accessory	Y	Y					Sec-SpI
687	809_00700	hypothetical protein	Accessory	Y	Y					Sec-SpI
688	809_00800	hypothetical protein	Accessory	Y	Y				1	Sec-SpI
689	809_01303	Shikimate dehydrogenase	Accessory	Y	Y					Sec-SpI
690	809_01783	Extracellular basic protease precursor	Accessory	Y	Y					Sec-SpI
691	809_01820	hypothetical protein	Accessory	Y	Y				1	Sec-SpI
692	809_02120	hypothetical protein	Accessory	Y	Y				1	Sec-SpI
693	809_02142	N-acetylmuramoyl-L-alanine amidase	Accessory	Y	Y				1	Sec-SpI
694	809_02228	hypothetical protein	Accessory	Y	Y				1	Sec-SpI
695	809_02242	hypothetical protein	Accessory	Y	Y				1	Sec-SpI
696	BRAD22_00188	hypothetical protein	Accessory	Y	Y					Sec-SpI
697	BRAD22_00249	Iron deficiency-induced protein A precursor	Accessory	Y	Y				1	Sec-SpI
698	BRAD22_00252	Alkaline phosphatase synthesis sensor protein PhoR	Accessory	Y	Y				1	Sec-SpI
699	BRAD22_00304	Diacylglycerol acyltransferase/mycolyltransferase Ag85A precursor	Accessory	Y	Y				1	Sec-SpI
700	BRAD22_00452	hypothetical protein	Accessory	Y	Y				1	Sec-SpI
701	BRAD22_00659	hypothetical protein	Accessory	Y	Y				1	Sec-SpI
702	BRAD22_00707	hypothetical protein	Accessory	Y	Y					Sec-SpI
703	BRAD22_01877	hypothetical protein	Accessory	Y	Y					Sec-SpI
704	BRAD22_01918	hypothetical protein	Accessory	Y	Y				1	Sec-SpI
705	BRAD22_02180	Serine-aspartate repeat-containing protein D precursor	Accessory	Y	Y					Sec-SpI
706	0102_00093	Alpha-amylase precursor	Accessory	Y	Y					Sec-SpI
707	0102_00102	Trypsin	Accessory	Y	Y					Sec-SpI
708	0102_00103	LGFP repeat protein	Accessory	Y	Y					Sec-SpI
709	0102_00218	Diphtheria toxin, C domain	Accessory	Y	Y					Sec-SpI
710	0102_00635	putative peptidase precursor	Accessory	Y	Y					Sec-SpI
711	2590_00672	hypothetical protein	Accessory	Y	Y				1	Sec-SpI
712	2590_00852	hypothetical protein	Accessory	Y	Y				1	Sec-SpI
713	2590_01053	hypothetical protein	Accessory	Y	Y				3	Sec-SpI
714	2590_01055	Cutinase	Accessory	Y	Y					Sec-SpI
715	2590_01426	hypothetical protein	Accessory	Y	Y					Sec-SpI
716	BRAD-2649_00897	hypothetical protein	Accessory	Y	Y				1	Sec-SpI
717	BRAD-2649_01344	hypothetical protein	Accessory	Y	Y				1	Sec-SpI
718	BRAD-2649_01372	Htaa	Accessory	Y	Y					Sec-SpI
719	BRAD-2649_02265	hypothetical protein	Accessory	Y	Y				1	Sec-SpI
720	BRAD-2649_02271	Htaa	Accessory	Y	Y					Sec-SpI
721	4940_00640	hypothetical protein	Accessory	Y	Y					Sec-SpI
722	4940_01183	hypothetical protein	Accessory	Y	Y				1	Sec-SpI
723	131001_01328	hypothetical protein	Accessory	Y	Y					Sec-SpI
724	131001_02024	Fimbrial subunit type 1 precursor	Accessory	Y	Y					Sec-SpI
725	131002_00065	META domain protein	Accessory	Y	Y				1	Sec-SpI
726	131002_00163	LGFP repeat protein	Accessory	Y	Y					Sec-SpI
727	131002_00676	hypothetical protein	Accessory	Y	Y					Sec-SpI
728	131002_00772	hypothetical protein	Accessory	Y	Y				1	Sec-SpI
729	131002_01229	Shikimate dehydrogenase	Accessory	Y	Y					Sec-SpI
730	131002_02055	hypothetical protein	Accessory	Y	Y				1	Sec-SpI
731	131002_02183	hypothetical protein	Accessory	Y	Y				1	Sec-SpI
732	210931_00246	hypothetical protein	Accessory	Y	Y					Sec-SpI
733	210931_00442	hypothetical protein	Accessory	Y	Y				1	Sec-SpI
734	210931_02095	hypothetical protein	Accessory	Y	Y					Sec-SpI

735	04-7514_00249	hypothetical protein	Accessory	Y	Y			Sec-SpI
736	04-7514_00254	hypothetical protein	Accessory	Y	Y			Sec-SpI
737	04-7514_00643	Htaa	Accessory	Y	Y			Sec-SpI
738	04-7514_00651	putative peptidase precursor	Accessory	Y	Y			Sec-SpI
739	04-7514_01918	hypothetical protein	Accessory	Y	Y		1	Sec-SpI
740	04-7514_02223	hypothetical protein	Accessory	Y	Y		1	Sec-SpI
741	04-7514_02246	N-acetylmuramoyl-L-alanine amidase	Accessory	Y	Y		1	Sec-SpI
742	FRC11_00091	hypothetical protein	Accessory	Y	Y			Sec-SpI
743	FRC11_00093	hypothetical protein	Accessory	Y	Y		1	Sec-SpI
744	FRC11_00681	hypothetical protein	Accessory	Y	Y			Sec-SpI
745	FRC11_02066	hypothetical protein	Accessory	Y	Y		1	Sec-SpI
746	LSPQ-04227_00237	hypothetical protein	Accessory	Y	Y			Sec-SpI
747	LSPQ-04227_00302	hypothetical protein	Accessory	Y	Y		1	Sec-SpI
748	LSPQ-04227_00480	hypothetical protein	Accessory	Y	Y			Sec-SpI
749	LSPQ-04227_00513	Thermostable alkaline protease precursor	Accessory	Y	Y		1	Sec-SpI
750	LSPQ-04227_02278	hypothetical protein	Accessory	Y	Y		1	Sec-SpI
751	LSPQ-04228_00209	Sialidase precursor	Accessory	Y	Y		1	Sec-SpI
752	LSPQ-04228_00225	Thermostable alkaline protease precursor	Accessory	Y	Y		1	Sec-SpI
753	NCTC12077_02353	von Willebrand factor type A domain protein	Accessory	Y	Y		1	Sec-SpI
754	NCTC12077_02490	hypothetical protein	Accessory	Y	Y		1	Sec-SpI
755	NCTC12077_00342	hypothetical protein	Accessory	Y	Y		1	Sec-SpI
756	809_00687	hypothetical protein	Accessory	Y	Y		1	Sec-SpI
757	809_00010	hypothetical protein	Accessory			2	3	TM
758	809_00013	hypothetical protein	Accessory			9	9	TM
759	809_00017	Ribose transport system permease protein RbsC	Accessory			9	9	TM
760	809_00019	hypothetical protein	Accessory			1	1	TM
761	809_00021	Rhomboid protease GluP	Accessory			7	6	TM
762	809_00025	hypothetical protein	Accessory			3	3	TM
763	809_00049	PP2C-family Ser/Thr phosphatase	Accessory			1	1	TM
764	809_00053	ABC-2 family transporter protein	Accessory			6	6	TM
765	809_00067	hypothetical protein	Accessory			12	12	TM
766	809_00073	hypothetical protein	Accessory			3	3	TM
767	809_00074	Major Facilitator Superfamily protein	Accessory			12	10	TM
768	809_00080	Thiol-disulfide oxidoreductase YkuV	Accessory			6	6	TM
769	809_00089	hypothetical protein	Accessory		Y	4	4	TM
770	809_00092	hypothetical protein	Accessory			2	2	TM
771	809_00094	hypothetical protein	Accessory		Y	2	2	TM
772	809_00107	Antiseptic resistance protein	Accessory			14	14	TM
773	809_00138	Threonine efflux protein	Accessory			6	5	TM
774	809_00166	phosphatidylglycerophosphatase B	Accessory			6	6	TM
775	809_00176	Sodium-dependent dicarboxylate transporter SdcS	Accessory			15	14	TM
776	809_00188	hypothetical protein	Accessory			2	2	TM
777	809_00198	Membrane protein YdfJ	Accessory		Y	11	12	TM
778	809_00226	Quinolone resistance protein NorB	Accessory			14	14	TM
779	809_00255	ABC-2 family transporter protein	Accessory			6	6	TM
780	809_00278	hypothetical protein	Accessory		Y	3	4	TM
781	809_00286	OPT oligopeptide transporter protein	Accessory			18	18	TM
782	809_00290	hypothetical protein	Accessory			2	2	TM
783	809_00307	hypothetical protein	Accessory		Y	1	2	TM
784	809_00323	hypothetical protein	Accessory			3	3	TM
785	809_00349	Fe(3+) dicitrate transport system permease protein FecD	Accessory			10	10	TM
786	809_00382	hypothetical protein	Accessory	Y		1	2	TM
787	809_00386	hypothetical protein	Accessory		Y	1	2	TM
788	809_00408	hypothetical protein	Accessory			3	3	TM
789	809_00410	hypothetical protein	Accessory			2	2	TM
790	809_00436	hypothetical protein	Accessory			2	2	TM
791	809_00465	ESX-1 secretion system protein eccB1	Accessory			1	1	TM
792	809_00468	hypothetical protein	Accessory			11	10	TM
793	809_00469	ESX-1 secretion system protein EccCa1	Accessory			2	1	TM
794	809_00470	hypothetical protein	Accessory			1	1	TM
795	809_00508	Bacterial membrane flanked domain protein	Accessory			4	3	TM
796	809_00516	hypothetical protein	Accessory			3	3	TM
797	809_00519	hypothetical protein	Accessory			3	3	TM
798	809_00561	hypothetical protein	Accessory			6	6	TM
799	809_00562	hypothetical protein	Accessory			6	6	TM
800	809_00568	putative ABC transporter permease protein	Accessory		Y	9	9	TM
801	809_00616	Voltage-gated potassium channel Kch	Accessory			2	2	TM

802	809_00629	hypothetical protein	Accessory		Y	3	5	TM
803	809_00632	Tyrocidine synthase 3	Accessory			7	7	TM
804	809_00652	hypothetical protein	Accessory	Y		1	1	TM
805	809_00662	hypothetical protein	Accessory			2	2	TM
806	809_00668	hypothetical protein	Accessory			1	1	TM
807	809_00671	Guanine/hypoxanthine permease PbuG	Accessory			12	13	TM
808	809_00684	hypothetical protein	Accessory			2	2	TM
809	809_00685	Cation/acetate symporter ActP	Accessory			13	13	TM
810	809_00689	Glutathione-regulated potassium-efflux system protein KefC	Accessory		Y	13	11	TM
811	809_00691	Multidrug resistance protein NorM	Accessory			11	11	TM
812	809_00718	hypothetical protein	Accessory			10	10	TM
813	809_00725	Arginine transport system permease protein ArtQ	Accessory			6	6	TM
814	809_00741	Large-conductance mechanosensitive channel	Accessory			2	2	TM
815	809_00747	hypothetical protein	Accessory			3	3	TM
816	809_00785	C4-dicarboxylate transporter/malic acid transport protein	Accessory			9	9	TM
817	809_00802	hypothetical protein	Accessory			1	1	TM
818	809_00828	putative ABC transporter ATP-binding protein	Accessory			5	4	TM
819	809_00829	putative ABC transporter ATP-binding protein	Accessory			6	6	TM
820	809_00831	Inner membrane protein YedI	Accessory			4	4	TM
821	809_00841	Oligopeptide transport system permease protein OppC	Accessory			6	6	TM
822	809_00845	outer membrane-specific lipoprotein transporter subunit LolE	Accessory			10	10	TM
823	809_00882	Putative multidrug export ATP-binding/permease protein	Accessory			10	11	TM
824	809_00895	hypothetical protein	Accessory			1	1	TM
825	809_00915	Glycolate permease GlcA	Accessory			13	13	TM
826	809_00927	hypothetical protein	Accessory			4	4	TM
827	809_00963	hypothetical protein	Accessory			4	3	TM
828	809_00972	DoxX	Accessory			5	5	TM
829	809_00975	putative MscS family protein YkuT	Accessory			4	3	TM
830	809_00997	hypothetical protein	Accessory			1	1	TM
831	809_00998	2-acyl-glycerophospho-ethanolamine acyltransferase	Accessory			11	10	TM
832	809_01103	hypothetical protein	Accessory			1	1	TM
833	809_01113	hypothetical protein	Accessory		Y	6	6	TM
834	809_01114	hypothetical protein	Accessory			6	6	TM
835	809_01120	hypothetical protein	Accessory			1	1	TM
836	809_01121	hypothetical protein	Accessory			2	2	TM
837	809_01127	phage T7 F exclusion suppressor FxsA	Accessory		Y	2	2	TM
838	809_01132	Proline-specific permease ProY	Accessory			12	12	TM
839	809_01140	Sec-independent protein translocase protein TatAd	Accessory			1	1	TM
840	809_01185	hypothetical protein	Accessory			1	1	TM
841	809_01191	hypothetical protein	Accessory			2	2	TM
842	809_01195	hypothetical protein	Accessory			4	4	TM
843	809_01196	hypothetical protein	Accessory			2	2	TM
844	809_01203	Phage-related minor tail protein	Accessory			2	2	TM
845	809_01219	hypothetical protein	Accessory			4	4	TM
846	809_01235	hypothetical protein	Accessory			1	1	TM
847	809_01251	hypothetical protein	Accessory			12	12	TM
848	809_01253	ABC-2 family transporter protein	Accessory			6	6	TM
849	809_01254	Heme A synthase	Accessory			7	8	TM
850	809_01255	Protoheme IX farnesyltransferase	Accessory			8	9	TM
851	809_01261	preprotein translocase subunit SecG	Accessory		Y	1	2	TM
852	809_01270	hypothetical protein	Accessory			2	2	TM
853	809_01302	Type IV leader peptidase family protein	Accessory			4	4	TM
854	809_01310	Putative neutral zinc metallopeptidase	Accessory			1	1	TM
855	809_01312	ABC-2 family transporter protein	Accessory			6	6	TM
856	809_01320	Peptidyl-prolyl cis-trans isomerase B	Accessory			1	1	TM
857	809_01325	preprotein translocase subunit SecF	Accessory			6	6	TM
858	809_01334	hypothetical protein	Accessory		Y	9	10	TM
859	809_01337	hypothetical protein	Accessory			1	1	TM
860	809_01345	hypothetical protein	Accessory	Y		1	2	TM
861	809_01370	hypothetical protein	Accessory			2	3	TM
862	809_01388	hypothetical protein	Accessory			1	1	TM
863	809_01456	hypothetical protein	Accessory			8	8	TM
864	809_01489	PTS system mannose-specific EIIBCA component	Accessory			10	9	TM
865	809_01553	hypothetical protein	Accessory		Y	1	2	TM
866	809_01562	Lipid II flippase FtsW	Accessory			10	8	TM
867	809_01576	hypothetical protein	Accessory			12	11	TM
868	809_01578	Serine/threonine-protein kinase PknL	Accessory			1	1	TM

869	809_01588	Arsenite oxidase subunit AioB precursor	Accessory			3	3	TM
870	809_01599	cobalamin synthase	Accessory			6	7	TM
871	809_01612	Fe(3+) dicitrate transport system permease protein FecD	Accessory		Y	9	8	TM
872	809_01621	hypothetical protein	Accessory			10	10	TM
873	809_01625	Na+/H+ antiporter family protein	Accessory		Y	11	11	TM
874	809_01693	hypothetical protein	Accessory			1	1	TM
875	809_01703	Glutathione transport system permease protein GsiC	Accessory	Y		6	6	TM
876	809_01710	FtsX-like permease family protein	Accessory	Y		10	10	TM
877	809_01724	ComEC family competence protein	Accessory			11	7	TM
878	809_01732	Inner membrane protein YbaN	Accessory	Y		3	2	TM
879	809_01741	hypothetical protein	Accessory			4	4	TM
880	809_01819	hypothetical protein	Accessory			2	2	TM
881	809_01821	Cytochrome c oxidase subunit 1	Accessory			12	12	TM
882	809_01840	camphor resistance protein CrcB	Accessory		Y	3	4	TM
883	809_01841	Putative fluoride ion transporter CrcB	Accessory		Y	3	4	TM
884	809_01859	Cytochrome bd-I ubiquinol oxidase subunit 1	Accessory			9	9	TM
885	809_01861	ATP-binding/permease protein CydD	Accessory			6	5	TM
886	809_01869	Phosphate transport system permease protein PstC	Accessory			6	6	TM
887	809_01930	Multidrug resistance protein stp	Accessory			14	14	TM
888	809_01960	Linear gramicidin synthase subunit D	Accessory			6	5	TM
889	809_02002	hypothetical protein	Accessory		Y	1	2	TM
890	809_02003	hypothetical protein	Accessory		Y	7	8	TM
891	809_02009	Cytochrome c biogenesis protein CcsA	Accessory			8	8	TM
892	809_02010	Cytochrome c biogenesis protein CcsB	Accessory			4	4	TM
893	809_02011	Tetratricopeptide repeat protein	Accessory			1	1	TM
894	809_02012	Cytochrome c-552 precursor	Accessory			1	1	TM
895	809_02013	Cytochrome c-type protein NrfH	Accessory			1	1	TM
896	809_02018	hypothetical protein	Accessory			4	4	TM
897	809_02019	hypothetical protein	Accessory			6	6	TM
898	809_02021	hypothetical protein	Accessory			2	2	TM
899	809_02032	hypothetical protein	Accessory			7	7	TM
900	809_02035	Sortase family protein	Accessory			2	2	TM
901	809_02037	Sortase family protein	Accessory			2	2	TM
902	809_02046	CAAX amino terminal protease self- immunity	Accessory			6	6	TM
903	809_02047	hypothetical protein	Accessory			4	3	TM
904	809_02049	Acyltransferase family protein	Accessory			10	10	TM
905	809_02050	Macrolide export ATP-binding/permease protein MacB	Accessory			4	4	TM
906	809_02067	pheromone autoinducer 2 transporter	Accessory			8	8	TM
907	809_02089	YibE/F-like protein	Accessory			8	8	TM
908	809_02092	hypothetical protein	Accessory			6	6	TM
909	809_02099	Iron import ATP-binding/permease protein IrtB	Accessory			5	5	TM
910	809_02100	Iron import ATP-binding/permease protein IrtA	Accessory			6	5	TM
911	809_02102	Energy-coupling factor transporter transmembrane protein EcfT	Accessory	Y		5	5	TM
912	809_02103	hypothetical protein	Accessory			1	1	TM
913	809_02104	hypothetical protein	Accessory			4	4	TM
914	809_02135	Putative undecaprenyl-diphosphatase YbjG	Accessory			5	3	TM
915	809_02154	Transcriptional regulator LytR	Accessory			1	1	TM
916	809_02157	Amino-acid carrier protein AlsT	Accessory			11	10	TM
917	809_02171	Polyprenol-phosphate-mannose-dependent alpha-(1-2)-phosphatidylinositol mannoside mannosyltransferase	Accessory			12	13	TM
918	809_02197	Copper-exporting P-type ATPase A	Accessory			8	8	TM
919	809_02205	hypothetical protein	Accessory		Y	1	2	TM
920	809_02206	hypothetical protein	Accessory			10	9	TM
921	809_02207	Penicillin-binding protein 1A	Accessory			1	1	TM
922	809_02212	hypothetical protein	Accessory			3	3	TM
923	809_02214	Inner membrane protein YqjA	Accessory			4	4	TM
924	809_02220	ABC-2 family transporter protein	Accessory			6	6	TM
925	809_02221	hypothetical protein	Accessory			5	5	TM
926	809_02224	FtsX-like permease family protein	Accessory			4	4	TM
927	809_02226	Sensor histidine kinase LiaS	Accessory			5	5	TM
928	809_02229	VanZ like family protein	Accessory			5	5	TM
929	809_02239	Oligopeptide transport ATP-binding protein OppD	Accessory			5	5	TM
930	809_02243	Tyrosine-specific transport protein	Accessory			11	11	TM
931	809_02248	putative amino acid permease YhdG	Accessory			12	12	TM
932	809_02258	Aerobic C4-dicarboxylate transport protein	Accessory		Y	9	10	TM
933	809_02269	Sodium Bile acid symporter family protein	Accessory			9	9	TM
934	809_02271	Branched-chain amino acid transport protein (AzlD)	Accessory			4	4	TM
935	809_02272	Inner membrane protein YgaZ	Accessory			6	4	TM

936	809_02276	hypothetical protein	Accessory			2	1	TM
937	809_02277	putative peptidoglycan biosynthesis protein MviN	Accessory			15	15	TM
938	BRAD22_00057	hypothetical protein	Accessory			4	3	TM
939	BRAD22_00061	Sensor histidine kinase DesK	Accessory			5	5	TM
940	BRAD22_00062	CAAX amino terminal protease self- immunity	Accessory			8	8	TM
941	BRAD22_00192	hypothetical protein	Accessory			1	1	TM
942	BRAD22_00226	Quinolone resistance protein NorB	Accessory			14	14	TM
943	BRAD22_00248	hypothetical protein	Accessory			14	12	TM
944	BRAD22_00250	Glycerol-3-phosphate transporter	Accessory			12	12	TM
945	BRAD22_00504	hypothetical protein	Accessory			6	6	TM
946	BRAD22_00528	hypothetical protein	Accessory			1	1	TM
947	BRAD22_00637	hypothetical protein	Accessory		Y	3	4	TM
948	BRAD22_00710	hypothetical protein	Accessory			1	1	TM
949	BRAD22_00711	hypothetical protein	Accessory			1	1	TM
950	BRAD22_01203	hypothetical protein	Accessory			4	4	TM
951	BRAD22_01736	hypothetical protein	Accessory			4	4	TM
952	BRAD22_01785	hypothetical protein	Accessory			1	1	TM
953	BRAD22_01867	hypothetical protein	Accessory			1	1	TM
954	BRAD22_01876	hypothetical protein	Accessory	Y		1	1	TM
955	BRAD22_01884	hypothetical protein	Accessory			6	5	TM
956	BRAD22_01919	Cytochrome c oxidase subunit 1	Accessory			12	12	TM
957	BRAD22_01930	hypothetical protein	Accessory			1	1	TM
958	BRAD22_02014	hypothetical protein	Accessory			4	4	TM
959	BRAD22_02015	hypothetical protein	Accessory			2	2	TM
960	BRAD22_02043	SPFH domain / Band 7 family protein	Accessory			2	2	TM
961	BRAD22_02055	hypothetical protein	Accessory			1	1	TM
962	BRAD22_02063	hypothetical protein	Accessory			1	1	TM
963	BRAD22_02093	hypothetical protein	Accessory			1	1	TM
964	BRAD22_02162	hypothetical protein	Accessory			1	1	TM
965	BRAD22_02193	Sortase family protein	Accessory			2	2	TM
966	BRAD22_02202	Macrolide export ATP-binding/permease protein MacB	Accessory			4	4	TM
967	BRAD22_02309	Amino-acid carrier protein AlsT	Accessory			11	10	TM
968	BRAD22_02310	O-acetyltransferase OatA	Accessory			11	9	TM
969	BRAD22_02387	VanZ like family protein	Accessory			5	5	TM
970	BRAD22_02388	hypothetical protein	Accessory			3	3	TM
971	0102_00072	Major Facilitator Superfamily protein	Accessory			10	8	TM
972	0102_00092	hypothetical protein	Accessory			2	2	TM
973	0102_00098	Antiseptic resistance protein	Accessory			9	8	TM
974	0102_00106	hypothetical protein	Accessory			4	4	TM
975	0102_00179	hypothetical protein	Accessory			1	1	TM
976	0102_00215	hypothetical protein	Accessory			2	2	TM
977	0102_00216	hypothetical protein	Accessory			4	4	TM
978	0102_00217	hypothetical protein	Accessory			1	1	TM
979	0102_00294	hypothetical protein	Accessory			1	1	TM
980	0102_00504	ESX-1 secretion system protein eccB1	Accessory			1	1	TM
981	0102_00508	hypothetical protein	Accessory			2	1	TM
982	0102_00510	hypothetical protein	Accessory			1	1	TM
983	0102_00565	hypothetical protein	Accessory			1	1	TM
984	0102_01097	putative type I restriction enzymeP M protein	Accessory			1	1	TM
985	0102_01195	hypothetical protein	Accessory			3	3	TM
986	0102_01205	hypothetical protein	Accessory			1	1	TM
987	0102_01298	hypothetical protein	Accessory			4	4	TM
988	0102_01342	hypothetical protein	Accessory			2	2	TM
989	0102_01722	hypothetical protein	Accessory			6	6	TM
990	0102_01726	Na+/H+ antiporter family protein	Accessory		Y	11	11	TM
991	0102_01935	hypothetical protein	Accessory			1	1	TM
992	0102_02133	hypothetical protein	Accessory			7	7	TM
993	0102_02289	hypothetical protein	Accessory			10	9	TM
994	0102_02296	Copper-exporting P-type ATPase A	Accessory			8	8	TM
995	2590_00457	hypothetical protein	Accessory			1	1	TM
996	2590_00973	hypothetical protein	Accessory			4	4	TM
997	2590_00974	hypothetical protein	Accessory		Y	3	2	TM
998	2590_00989	putative ABC transporter permease protein	Accessory		Y	9	9	TM
999	2590_01054	hypothetical protein	Accessory			1	1	TM
1000	2590_01057	TrbC/VIRB2 family protein	Accessory			2	2	TM
1001	2590_01058	hypothetical protein	Accessory			2	2	TM
1002	2590_01062	hypothetical protein	Accessory			2	2	TM

1003	2590_01067	TraM recognition site of TraD and TraG	Accessory			2	2	TM
1004	2590_01069	hypothetical protein	Accessory			1	1	TM
1005	2590_01481	Hemin transport system permease protein HmuU	Accessory		Y	7	8	TM
1006	2590_01489	hypothetical protein	Accessory			2	2	TM
1007	2590_01533	Threonine efflux protein	Accessory			6	5	TM
1008	2590_01557	hypothetical protein	Accessory			4	4	TM
1009	2590_01634	hypothetical protein	Accessory			1	1	TM
1010	2590_01636	hypothetical protein	Accessory			1	1	TM
1011	2590_01704	putative amino acid permease YhdG	Accessory			12	12	TM
1012	2590_01873	hypothetical protein	Accessory			1	1	TM
1013	2590_02237	hypothetical protein	Accessory			1	1	TM
1014	BRAD-2649_00127	hypothetical protein	Accessory			5	5	TM
1015	BRAD-2649_00447	hypothetical protein	Accessory			2	1	TM
1016	BRAD-2649_00473	Tyrocidine synthase 3	Accessory			6	7	TM
1017	BRAD-2649_00857	Putative pseudouridine transporter	Accessory			2	2	TM
1018	BRAD-2649_00927	YibE/F-like protein	Accessory			8	8	TM
1019	BRAD-2649_00966	Macrolide export ATP-binding/permease protein MacB	Accessory			4	4	TM
1020	BRAD-2649_00969	hypothetical protein	Accessory			4	4	TM
1021	BRAD-2649_00973	Antiseptic resistance protein	Accessory			14	12	TM
1022	BRAD-2649_00992	Sortase family protein	Accessory			2	2	TM
1023	BRAD-2649_00994	Sortase family protein	Accessory			2	2	TM
1024	BRAD-2649_00997	hypothetical protein	Accessory			2	2	TM
1025	BRAD-2649_00998	hypothetical protein	Accessory			1	1	TM
1026	BRAD-2649_01012	hypothetical protein	Accessory			4	4	TM
1027	BRAD-2649_01063	hypothetical protein	Accessory			1	1	TM
1028	BRAD-2649_01064	Linear gramicidin synthase subunit D	Accessory			6	5	TM
1029	BRAD-2649_01122	hypothetical protein	Accessory			1	1	TM
1030	BRAD-2649_01136	Sodium-dependent dicarboxylate transporter SdcS	Accessory	Y		10	10	TM
1031	BRAD-2649_01231	hypothetical protein	Accessory			3	2	TM
1032	BRAD-2649_01343	tryptophan permease	Accessory			7	7	TM
1033	BRAD-2649_01356	hypothetical protein	Accessory			3	3	TM
1034	BRAD-2649_01371	hypothetical protein	Accessory			1	1	TM
1035	BRAD-2649_01566	Inner membrane protein YedI	Accessory			4	4	TM
1036	BRAD-2649_01648	hypothetical protein	Accessory			2	2	TM
1037	BRAD-2649_01816	TM2 domain protein	Accessory			2	1	TM
1038	BRAD-2649_01826	hypothetical protein	Accessory			1	1	TM
1039	BRAD-2649_01841	hypothetical protein	Accessory			2	2	TM
1040	BRAD-2649_02264	DNA-binding transcriptional activator PspC	Accessory			1	1	TM
1041	BRAD-2649_02270	hypothetical protein	Accessory			1	1	TM
1042	BRAD-2649_02272	Htaa	Accessory			1	1	TM
1043	BRAD-2649_02322	camphor resistance protein CrcB	Accessory		Y	3	4	TM
1044	BRAD-2649_02328	Phage-related minor tail protein	Accessory			2	2	TM
1045	4940_01304	PP2C-family Ser/Thr phosphatase	Accessory			1	1	TM
1046	4940_01546	hypothetical protein	Accessory			3	3	TM
1047	4940_01622	hypothetical protein	Accessory			7	7	TM
1048	4940_01624	hypothetical protein	Accessory	Y		1	1	TM
1049	4940_01940	hypothetical protein	Accessory			1	1	TM
1050	4940_02202	Arsenite oxidase subunit AioB precursor	Accessory			3	3	TM
1051	131001_00093	hypothetical protein	Accessory			2	2	TM
1052	131001_00170	hypothetical protein	Accessory			1	1	TM
1053	131001_00552	Major Facilitator Superfamily protein	Accessory			12	12	TM
1054	131001_01330	hypothetical protein	Accessory			5	5	TM
1055	131001_01442	hypothetical protein	Accessory			8	8	TM
1056	131001_01813	hypothetical protein	Accessory			1	1	TM
1057	131001_01876	Ktr system potassium uptake protein B	Accessory			2	2	TM
1058	131001_01893	hypothetical protein	Accessory			3	2	TM
1059	131002_00013	hypothetical protein	Accessory			9	9	TM
1060	131002_00067	hypothetical protein	Accessory			12	12	TM
1061	131002_00072	hypothetical protein	Accessory			3	3	TM
1062	131002_00188	Membrane protein YdfJ	Accessory		Y	11	12	TM
1063	131002_00264	hypothetical protein	Accessory		Y	3	3	TM
1064	131002_00293	hypothetical protein	Accessory		Y	1	2	TM
1065	131002_00309	hypothetical protein	Accessory			3	3	TM
1066	131002_00393	hypothetical protein	Accessory			3	3	TM
1067	131002_00395	hypothetical protein	Accessory			2	2	TM
1068	131002_00448	ESX-1 secretion system protein eccB1	Accessory			1	1	TM
1069	131002_00451	hypothetical protein	Accessory			11	8	TM

1070	131002_00452	ESX-1 secretion system protein EccCa1	Accessory			2	1	TM
1071	131002_00453	hypothetical protein	Accessory			1	1	TM
1072	131002_00493	Bacterial membrane flanked domain protein	Accessory			4	3	TM
1073	131002_00720	hypothetical protein	Accessory			3	3	TM
1074	131002_00774	hypothetical protein	Accessory			1	1	TM
1075	131002_00813	Oligopeptide transport system permease protein OppC	Accessory			6	6	TM
1076	131002_00817	FtsX-like permease family protein	Accessory			10	10	TM
1077	131002_00867	hypothetical protein	Accessory			1	1	TM
1078	131002_00935	hypothetical protein	Accessory			4	4	TM
1079	131002_00944	DoxX	Accessory			5	5	TM
1080	131002_00947	putative MscS family protein YkuT	Accessory			4	3	TM
1081	131002_00951	hypothetical protein	Accessory			1	1	TM
1082	131002_00974	hypothetical protein	Accessory			2	2	TM
1083	131002_01098	hypothetical protein	Accessory			2	2	TM
1084	131002_01162	hypothetical protein	Accessory			1	1	TM
1085	131002_01228	Type IV leader peptidase family protein	Accessory			4	4	TM
1086	131002_01272	hypothetical protein	Accessory	Y		1	2	TM
1087	131002_01273	EamA-like transporter family protein	Accessory			8	8	TM
1088	131002_01315	hypothetical protein	Accessory			1	1	TM
1089	131002_01384	hypothetical protein	Accessory			8	8	TM
1090	131002_01416	PTS system mannose-specific EIIBCA component	Accessory			10	9	TM
1091	131002_01528	cobalamin synthase	Accessory			6	8	TM
1092	131002_01542	Ferric enterobactin transport system permease protein FepG	Accessory		Y	3	4	TM
1093	131002_01654	ComEC family competence protein	Accessory		Y	7	7	TM
1094	131002_01668	hypothetical protein	Accessory			4	4	TM
1095	131002_01768	camphor resistance protein CrcB	Accessory		Y	3	4	TM
1096	131002_01769	camphor resistance protein CrcB	Accessory			4	4	TM
1097	131002_01790	Cytochrome bd-I ubiquinol oxidase subunit 1	Accessory			9	9	TM
1098	131002_01792	ATP-binding/permease protein CydD	Accessory			5	5	TM
1099	131002_01936	hypothetical protein	Accessory			8	8	TM
1100	131002_01952	ABC-2 family transporter protein	Accessory			6	6	TM
1101	131002_02028	hypothetical protein	Accessory			6	6	TM
1102	131002_02070	Putative undecaprenyl-diphosphatase YbjG	Accessory			5	4	TM
1103	131002_02159	hypothetical protein	Accessory			5	5	TM
1104	131002_02168	VanZ like family protein	Accessory			5	5	TM
1105	131002_02184	Tyrosine-specific transport protein	Accessory			11	11	TM
1106	131002_02212	Branched-chain amino acid transport protein (AzlD)	Accessory			4	4	TM
1107	210931_00245	hypothetical protein	Accessory			3	3	TM
1108	210931_01917	hypothetical protein	Accessory			1	1	TM
1109	210931_01986	hypothetical protein	Accessory			1	1	TM
1110	210931_02088	pheromone autoinducer 2 transporter	Accessory			8	8	TM
1111	210931_02094	hypothetical protein	Accessory			1	1	TM
1112	210931_02275	Glutathione transport system permease protein GsiC	Accessory			2	2	TM
1113	04-3911_00509	hypothetical protein	Accessory			2	1	TM
1114	04-3911_00884	hypothetical protein	Accessory			3	3	TM
1115	04-3911_00923	Putative multidrug export ATP-binding/permease protein	Accessory			10	11	TM
1116	04-3911_01978	hypothetical protein	Accessory			2	1	TM
1117	04-7514_00073	hypothetical protein	Accessory			1	1	TM
1118	04-7514_00075	Major Facilitator Superfamily protein	Accessory			2	2	TM
1119	04-7514_00217	hypothetical protein	Accessory			4	3	TM
1120	04-7514_00332	OPT oligopeptide transporter protein	Accessory			18	18	TM
1121	04-7514_00334	hypothetical protein	Accessory			4	4	TM
1122	04-7514_00337	hypothetical protein	Accessory			2	2	TM
1123	04-7514_00633	hypothetical protein	Accessory			3	3	TM
1124	04-7514_00644	hypothetical protein	Accessory			1	1	TM
1125	04-7514_00680	hypothetical protein	Accessory			2	2	TM
1126	04-7514_00775	hypothetical protein	Accessory			2	2	TM
1127	04-7514_00835	hypothetical protein	Accessory			9	8	TM
1128	04-7514_00850	hypothetical protein	Accessory			1	1	TM
1129	04-7514_00855	Periplasmic pH-dependent serine endoprotease DegQ precursor	Accessory			1	1	TM
1130	04-7514_01036	Glycolate permease GlcA	Accessory			13	13	TM
1131	04-7514_01087	hypothetical protein	Accessory			4	3	TM
1132	04-7514_01096	DoxX	Accessory			5	5	TM
1133	04-7514_01119	hypothetical protein	Accessory			1	1	TM
1134	04-7514_01249	hypothetical protein	Accessory			2	2	TM
1135	04-7514_01395	ABC-2 family transporter protein	Accessory			5	4	TM
1136	04-7514_01432	hypothetical protein	Accessory			1	2	TM

1137	04-7514_01451	hypothetical protein	Accessory			2	3	TM
1138	04-7514_01470	hypothetical protein	Accessory			1	1	TM
1139	04-7514_01663	hypothetical protein	Accessory			6	5	TM
1140	04-7514_01836	hypothetical protein	Accessory			4	4	TM
1141	04-7514_01931	hypothetical protein	Accessory			1	1	TM
1142	04-7514_01971	Phosphate transport system permease protein PstC	Accessory			1	1	TM
1143	04-7514_02010	hypothetical protein	Accessory	Y		1	1	TM
1144	04-7514_02034	Multidrug resistance protein stp	Accessory			14	14	TM
1145	04-7514_02038	hypothetical protein	Accessory			1	1	TM
1146	04-7514_02122	hypothetical protein	Accessory			1	1	TM
1147	04-7514_02137	hypothetical protein	Accessory			1	1	TM
1148	04-7514_02203	hypothetical protein	Accessory			5	5	TM
1149	04-7514_02204	Iron import ATP-binding/permease protein IrtA	Accessory			6	5	TM
1150	04-7514_02238	Putative undecaprenyl-diphosphatase YbjG	Accessory			5	4	TM
1151	04-7514_02273	Polyprenol-phosphate-mannose-dependent alpha-(1-2)-phosphatidylinositol mannoside mannosyltransferase	Accessory			2	2	TM
1152	04-7514_02319	hypothetical protein	Accessory			1	1	TM
1153	04-7514_02390	hypothetical protein	Accessory			1	1	TM
1154	210932_00804	putative ABC transporter ATP-binding protein	Accessory			6	6	TM
1155	210932_01993	Cytochrome c biogenesis protein Ccs1	Accessory			4	4	TM
1156	FRC11_00071	hypothetical protein	Accessory			3	3	TM
1157	FRC11_00092	hypothetical protein	Accessory			1	1	TM
1158	FRC11_00419	hypothetical protein	Accessory			2	2	TM
1159	FRC11_00640	hypothetical protein	Accessory			3	3	TM
1160	FRC11_01048	hypothetical protein	Accessory			3	3	TM
1161	FRC11_01101	hypothetical protein	Accessory		Y	6	6	TM
1162	FRC11_01273	hypothetical protein	Accessory		Y	9	10	TM
1163	FRC11_01629	hypothetical protein	Accessory			1	1	TM
1164	FRC11_01667	ComEC family competence protein	Accessory		Y	7	5	TM
1165	FRC11_01681	hypothetical protein	Accessory			4	3	TM
1166	FRC11_01760	hypothetical protein	Accessory			2	2	TM
1167	FRC11_01782	camphor resistance protein CrcB	Accessory			4	4	TM
1168	FRC11_01795	hypothetical protein	Accessory			2	2	TM
1169	FRC11_02134	hypothetical protein	Accessory			3	3	TM
1170	FRC11_02188	tryptophan permease	Accessory			6	6	TM
1171	FRC58_00328	Phage-related minor tail protein	Accessory			4	4	TM
1172	FRC58_00335	hypothetical protein	Accessory			2	2	TM
1173	FRC58_01658	hypothetical protein	Accessory			1	1	TM
1174	FRC58_01659	hypothetical protein	Accessory		Y	3	4	TM
1175	FRC58_01667	Phage-related minor tail protein	Accessory			6	4	TM
1176	FRC58_01690	hypothetical protein	Accessory			2	2	TM
1177	KZN-2016-48390_00762	hypothetical protein	Accessory			2	1	TM
1178	KZN-2016-48390_00764	CDP-diacylglycerol--inositol 3-phosphatidyltransferase	Accessory			5	3	TM
1179	KZN-2016-48390_01070	hypothetical protein	Accessory			2	2	TM
1180	LSPQ-04227_00013	putative peptidoglycan biosynthesis protein MviN	Accessory			1	1	TM
1181	LSPQ-04227_00044	Ribose transport system permease protein RbsC	Accessory		Y	3	4	TM
1182	LSPQ-04227_00101	hypothetical protein	Accessory			3	2	TM
1183	LSPQ-04227_00108	Thiol-disulfide oxidoreductase YkuV	Accessory			6	6	TM
1184	LSPQ-04227_00224	Membrane protein YdfJ	Accessory			2	2	TM
1185	LSPQ-04227_00259	Serine-aspartate repeat-containing protein D precursor	Accessory			1	1	TM
1186	LSPQ-04227_00264	ABC-2 type transporter	Accessory			7	7	TM
1187	LSPQ-04227_00287	hypothetical protein	Accessory			2	2	TM
1188	LSPQ-04227_00288	hypothetical protein	Accessory			1	1	TM
1189	LSPQ-04227_00323	hypothetical protein	Accessory			5	5	TM
1190	LSPQ-04227_00673	hypothetical protein	Accessory			1	1	TM
1191	LSPQ-04227_00691	hypothetical protein	Accessory			2	2	TM
1192	LSPQ-04227_00809	hypothetical protein	Accessory			2	2	TM
1193	LSPQ-04227_00906	hypothetical protein	Accessory			1	2	TM
1194	LSPQ-04227_00951	Lipid II flippase FtsW	Accessory		Y	3	4	TM
1195	LSPQ-04227_01233	hypothetical protein	Accessory			2	2	TM
1196	LSPQ-04227_01253	hypothetical protein	Accessory			1	1	TM
1197	LSPQ-04227_01278	hypothetical protein	Accessory			1	1	TM
1198	LSPQ-04227_01527	Multidrug resistance protein 3	Accessory			6	6	TM
1199	LSPQ-04227_01619	Arginine transport system permease protein ArtQ	Accessory			6	6	TM
1200	LSPQ-04227_01656	Multidrug resistance protein NorM	Accessory		Y	5	6	TM
1201	LSPQ-04227_01680	hypothetical protein	Accessory			2	2	TM
1202	LSPQ-04227_01826	hypothetical protein	Accessory			2	2	TM
1203	LSPQ-04227_01869	Sec-independent protein translocase protein TatC	Accessory			1	1	TM

1204	LSPQ-04227_01877	Proline-specific permease ProY	Accessory					12	12	TM
1205	LSPQ-04227_01882	phage T7 F exclusion suppressor FxsA	Accessory		Y			2	3	TM
1206	LSPQ-04227_01961	Cytochrome bd-II ubiquinol oxidase subunit 1	Accessory					1	1	TM
1207	LSPQ-04227_02001	Phosphoribosylamine--glycine ligase	Accessory					2	1	TM
1208	LSPQ-04227_02043	hypothetical protein	Accessory					2	2	TM
1209	LSPQ-04227_02118	Disulfide bond formation protein DsbB	Accessory					5	5	TM
1210	LSPQ-04227_02136	hypothetical protein	Accessory					1	1	TM
1211	LSPQ-04227_02163	hypothetical protein	Accessory					6	6	TM
1212	LSPQ-04227_02171	CAAX amino terminal protease self- immunity	Accessory					3	4	TM
1213	LSPQ-04227_02200	hypothetical protein	Accessory					2	2	TM
1214	LSPQ-04227_02265	hypothetical protein	Accessory					6	7	TM
1215	LSPQ-04227_02276	Hemin transport system permease protein HmuU	Accessory					9	8	TM
1216	LSPQ-04227_02292	Transcriptional regulator LytR	Accessory					1	1	TM
1217	LSPQ-04227_02326	hypothetical protein	Accessory					2	2	TM
1218	LSPQ-04227_02411	Tetracycline resistance protein, class C	Accessory					3	3	TM
1219	LSPQ-04227_02415	Tetracycline resistance protein, class C	Accessory		Y			7	7	TM
1220	LSPQ-04228_00144	hypothetical protein	Accessory					1	1	TM
1221	LSPQ-04228_00277	Manganese transport system membrane protein MntB	Accessory					4	4	TM
1222	LSPQ-04228_00298	hypothetical protein	Accessory					1	1	TM
1223	LSPQ-04228_00378	hypothetical protein	Accessory					1	1	TM
1224	LSPQ-04228_00586	hypothetical protein	Accessory					3	3	TM
1225	LSPQ-04228_00603	Glycolate permease GlcA	Accessory					5	5	TM
1226	LSPQ-04228_00660	GABA permease	Accessory					4	3	TM
1227	LSPQ-04228_00661	GABA permease	Accessory					2	3	TM
1228	LSPQ-04228_00666	GtrA-like protein	Accessory					1	1	TM
1229	LSPQ-04228_00745	C4-dicarboxylate transporter/malic acid transport protein	Accessory					9	9	TM
1230	LSPQ-04228_00930	hypothetical protein	Accessory					1	1	TM
1231	LSPQ-04228_00931	hypothetical protein	Accessory					1	2	TM
1232	LSPQ-04228_01079	Lipid II flippase FtsW	Accessory		Y			3	4	TM
1233	LSPQ-04228_01080	Lipid II flippase FtsW	Accessory					6	4	TM
1234	LSPQ-04228_01103	Acyltransferase family protein	Accessory					4	3	TM
1235	LSPQ-04228_01184	hypothetical protein	Accessory					2	3	TM
1236	LSPQ-04228_01192	hypothetical protein	Accessory					1	1	TM
1237	LSPQ-04228_01349	hypothetical protein	Accessory					2	2	TM
1238	LSPQ-04228_01357	hypothetical protein	Accessory					2	2	TM
1239	LSPQ-04228_01421	hypothetical protein	Accessory					1	1	TM
1240	LSPQ-04228_01492	Membrane protein YdfJ	Accessory		Y			5	6	TM
1241	LSPQ-04228_01786	SPFH domain / Band 7 family protein	Accessory					2	2	TM
1242	LSPQ-04228_01790	hypothetical protein	Accessory					2	2	TM
1243	LSPQ-04228_01947	hypothetical protein	Accessory					6	7	TM
1244	LSPQ-04228_01965	MMPL family protein	Accessory					1	1	TM
1245	LSPQ-04228_02067	hypothetical protein	Accessory					9	8	TM
1246	NCTC12077_00168	hypothetical protein	Accessory		Y			4	5	TM
1247	NCTC12077_00262	hypothetical protein	Accessory					3	3	TM
1248	NCTC12077_00490	Putative cryptic C4-dicarboxylate transporter DcuD	Accessory		Y			7	8	TM
1249	NCTC12077_00659	hypothetical protein	Accessory					1	1	TM
1250	NCTC12077_00664	hypothetical protein	Accessory					1	1	TM
1251	NCTC12077_01221	hypothetical protein	Accessory					1	1	TM
1252	NCTC12077_01924	Glycine betaine transporter OpuD	Accessory					12	12	TM
1253	NCTC12077_01928	Nickel transport system permease protein NikB	Accessory					3	3	TM
1254	NCTC12077_02481	Alpha-(1->3)-arabinofuranosyltransferase	Accessory					12	10	TM
1255	NCTC12077_02491	hypothetical protein	Accessory					2	2	TM
1256	LSPQ-04227_02242	hypothetical protein	Accessory					1	1	TM
1257	BRAD22_01900	Peptidoglycan-N-acetylglucosamine deacetylase	Accessory	Y		SpII	Lipoprotein	1	1	TM-Lipo
1258	809_00221	Chromosome partition protein Smc	Accessory	Y	Y			1	2	TM-Sec
1259	809_00252	Na(+)/H(+) antiporter subunit A	Accessory	Y	Y			23	23	TM-Sec
1260	809_00385	hypothetical protein	Accessory	Y	Y			1	1	TM-Sec
1261	809_00467	Thermostable alkaline protease precursor	Accessory	Y	Y			1	2	TM-Sec
1262	809_00524	Htaa	Accessory	Y	Y			1	1	TM-Sec
1263	809_00532	putative peptidase precursor	Accessory	Y	Y			1	1	TM-Sec
1264	809_00979	hypothetical protein	Accessory	Y	Y			1	1	TM-Sec
1265	809_01093	hypothetical protein	Accessory	Y	Y			2	3	TM-Sec
1266	809_01321	hypothetical protein	Accessory	Y	Y			1	1	TM-Sec
1267	809_01921	hypothetical protein	Accessory	Y	Y			1	2	TM-Sec
1268	809_01933	Chromosome partition protein Smc	Accessory	Y	Y			1	2	TM-Sec
1269	809_02041	Chromosome partition protein Smc	Accessory	Y	Y			1	1	TM-Sec
1270	809_02042	chromosome segregation protein	Accessory	Y	Y			1	1	TM-Sec

1271	809_02048	putative peptidase precursor	Accessory	Y	Y	1	1		TM-Sec
1272	809_02108	Alpha-(1->3)-arabinofuranosyltransferase	Accessory	Y	Y	12	9		TM-Sec
1273	809_02155	hypothetical protein	Accessory	Y	Y	1	1		TM-Sec
1274	809_02198	hypothetical protein	Accessory	Y	Y	1	1		TM-Sec
1275	809_02215	Htaa	Accessory	Y	Y	1	1		TM-Sec
1276	809_02219	VanZ like family protein	Accessory	Y	Y	4	5		TM-Sec
1277	BRAD22_00221	Chromosome partition protein Smc	Accessory	Y	Y	1	1		TM-Sec
1278	BRAD22_00533	Htaa	Accessory	Y	Y	1	1		TM-Sec
1279	BRAD22_01329	hypothetical protein	Accessory	Y	Y	1	2		TM-Sec
1280	BRAD22_02197	Chromosome partition protein Smc	Accessory	Y	Y	1	1		TM-Sec
1281	BRAD22_02198	chromosome segregation protein	Accessory	Y	Y	1	1		TM-Sec
1282	BRAD22_02307	hypothetical protein	Accessory	Y	Y	1	1		TM-Sec
1283	0102_00291	Na(+)/H(+) antiporter subunit A	Accessory	Y	Y	23	23		TM-Sec
1284	0102_00628	Htaa	Accessory	Y	Y	1	1		TM-Sec
1285	0102_01423	hypothetical protein	Accessory	Y	Y	1	1		TM-Sec
1286	0102_02144	chromosome segregation protein	Accessory	Y	Y	1	1		TM-Sec
1287	0102_02253	hypothetical protein	Accessory	Y	Y	1	1		TM-Sec
1288	0102_02302	hypothetical protein	Accessory	Y	Y	1	1		TM-Sec
1289	2590_00933	Htaa	Accessory	Y	Y	1	1		TM-Sec
1290	2590_01061	hypothetical protein	Accessory	Y	Y	6	8		TM-Sec
1291	2590_01736	Htaa	Accessory	Y	Y	1	1		TM-Sec
1292	2590_01753	hypothetical protein	Accessory	Y	Y	1	1		TM-Sec
1293	4940_00210	hypothetical protein	Accessory	Y	Y	1	2		TM-Sec
1294	4940_00482	hypothetical protein	Accessory	Y	Y	1	1		TM-Sec
1295	131001_00366	hypothetical protein	Accessory	Y	Y	1	2		TM-Sec
1296	131001_01814	hypothetical protein	Accessory	Y	Y	1	1		TM-Sec
1297	131001_02030	chromosome segregation protein	Accessory	Y	Y	1	1		TM-Sec
1298	131001_02201	Htaa	Accessory	Y	Y	1	1		TM-Sec
1299	131002_00370	hypothetical protein	Accessory	Y	Y	1	1		TM-Sec
1300	131002_00450	Thermostable alkaline protease precursor	Accessory	Y	Y	1	2		TM-Sec
1301	131002_00516	putative peptidase precursor	Accessory	Y	Y	1	1		TM-Sec
1302	131002_01249	hypothetical protein	Accessory	Y	Y	1	2		TM-Sec
1303	131002_01760	hypothetical protein	Accessory	Y	Y	1	2		TM-Sec
1304	131002_01856	hypothetical protein	Accessory	Y	Y	1	2		TM-Sec
1305	131002_01976	chromosome segregation protein	Accessory	Y	Y	1	1		TM-Sec
1306	131002_02090	hypothetical protein	Accessory	Y	Y	1	1		TM-Sec
1307	131002_02136	hypothetical protein	Accessory	Y	Y	1	1		TM-Sec
1308	210931_00522	Htaa	Accessory	Y	Y	1	1		TM-Sec
1309	210931_01269	hypothetical protein	Accessory	Y	Y	1	1		TM-Sec
1310	04-7514_00516	Thermostable alkaline protease precursor	Accessory	Y	Y	1	2		TM-Sec
1311	04-7514_02025	hypothetical protein	Accessory	Y	Y	1	2		TM-Sec
1312	04-7514_02039	Chromosome partition protein Smc	Accessory	Y	Y	1	2		TM-Sec
1313	04-7514_02259	hypothetical protein	Accessory	Y	Y	1	1		TM-Sec
1314	FRC11_00212	Chromosome partition protein Smc	Accessory	Y	Y	1	1		TM-Sec
1315	FRC11_01774	hypothetical protein	Accessory	Y	Y	1	2		TM-Sec
1316	FRC11_02142	hypothetical protein	Accessory	Y	Y	1	1		TM-Sec
1317	LSPQ-04227_02166	chromosome segregation protein	Accessory	Y	Y	1	1		TM-Sec
1318	LSPQ-04227_02239	Alpha-(1->3)-arabinofuranosyltransferase	Accessory	Y	Y	8	8		TM-Sec
1319	LSPQ-04228_01424	Na(+)/H(+) antiporter subunit A	Accessory	Y	Y	10	10		TM-Sec
1320	809_01832	hypothetical protein	Accessory	Y	Y	1	1		TM-Sec
1321	809_01833	hypothetical protein	Accessory	Y	Y	1	2		TM-Sec
1322	809_02034	hypothetical protein	Accessory		Y	1	2	sortaseA	LPXTG
1323	BRAD22_00230	Serine-aspartate repeat-containing protein D precursor	Accessory	Y		1	1	sortaseD	LPXTG
1324	809_00230	Serine-aspartate repeat-containing protein D precursor	Accessory	Y		1	1	sortaseD	LPXTG
1325	809_01831	hypothetical protein	Accessory	Y		2	2	sortaseD	LPXTG
1326	809_01906	hypothetical protein	Accessory	Y		2	2	sortaseD	LPXTG
1327	809_02033	hypothetical protein	Accessory	Y		2	2	sortaseA	LPXTG
1328	BRAD22_02192	Fimbrial subunit type 1 precursor	Accessory	Y		1	1	sortaseA	LPXTG
1329	0102_02134	hypothetical protein	Accessory	Y		2	2	sortaseA	LPXTG
1330	2590_02191	Serine-aspartate repeat-containing protein D precursor	Accessory	Y		1	1	sortaseD	LPXTG
1331	BRAD-2649_00472	hypothetical protein	Accessory	Y		2	2	sortaseD	LPXTG
1332	BRAD-2649_00995	hypothetical protein	Accessory		Y	1	2	sortaseA	LPXTG
1333	210931_01993	T surface-antigen of pili	Accessory		Y	1	2	sortaseD	LPXTG
1334	809_00022	hypothetical protein	Accessory	Y	Y	1	2	sortaseD	LPXTG
1335	809_00229	hypothetical protein	Accessory	Y	Y	1	1	sortaseD	LPXTG
1336	809_00236	T surface-antigen of pili	Accessory	Y	Y	1	2	sortaseD	LPXTG
1337	809_00380	hypothetical protein	Accessory	Y	Y	1	2	sortaseA	LPXTG

1338	809_00390	hypothetical protein	Accessory	Y	Y	2	2	sortaseD	LPXTG
1339	809_01193	hypothetical protein	Accessory	Y	Y	1	2	sortaseD	LPXTG
1340	809_01973	T surface-antigen of pili	Accessory	Y	Y	1	2	sortaseD	LPXTG
1341	809_02024	Serine-aspartate repeat-containing protein D precursor	Accessory	Y	Y	1	1	sortaseD	LPXTG
1342	809_02036	Fimbrial subunit type 1 precursor	Accessory	Y	Y	1	1	sortaseA	LPXTG
1343	809_02225	hypothetical protein	Accessory	Y	Y	1	2	sortaseD	LPXTG
1344	809_02230	hypothetical protein	Accessory	Y	Y	1	2	sortaseD	LPXTG
1345	BRAD22_01201	hypothetical protein	Accessory	Y	Y	1	2	sortaseD	LPXTG
1346	BRAD22_02007	hypothetical protein	Accessory	Y	Y	1	3	sortaseD	LPXTG
1347	BRAD22_02131	T surface-antigen of pili	Accessory	Y	Y	1	1	sortaseD	LPXTG
1348	BRAD22_02287	hypothetical protein	Accessory	Y	Y	1	2	sortaseD	LPXTG
1349	BRAD22_02383	hypothetical protein	Accessory	Y	Y	1	2	sortaseD	LPXTG
1350	0102_01296	hypothetical protein	Accessory	Y	Y	1	2	sortaseD	LPXTG
1351	0102_02124	Serine-aspartate repeat-containing protein D precursor	Accessory	Y	Y	1	1	sortaseD	LPXTG
1352	0102_02137	Fimbrial subunit type 1 precursor	Accessory	Y	Y	1	2	sortaseA	LPXTG
1353	2590_02003	Fimbrial subunit type 1 precursor	Accessory	Y	Y	1	1	sortaseA	LPXTG
1354	2590_02054	hypothetical protein	Accessory	Y	Y	1	2	sortaseD	LPXTG
1355	2590_02190	hypothetical protein	Accessory	Y	Y	1	1	sortaseD	LPXTG
1356	2590_02197	T surface-antigen of pili	Accessory	Y	Y	1	2	sortaseD	LPXTG
1357	BRAD-2649_00712	hypothetical protein	Accessory	Y	Y	2	2	sortaseD	LPXTG
1358	BRAD-2649_01051	T surface-antigen of pili	Accessory	Y	Y	1	2	sortaseD	LPXTG
1359	BRAD-2649_01069	hypothetical protein	Accessory	Y	Y	1	1	sortaseD	LPXTG
1360	BRAD-2649_01281	hypothetical protein	Accessory	Y	Y	1	2	sortaseD	LPXTG
1361	BRAD-2649_01355	hypothetical protein	Accessory	Y	Y	1	2	sortaseD	LPXTG
1362	4940_00509	hypothetical protein	Accessory	Y	Y	1	2	sortaseD	LPXTG
1363	131001_00364	hypothetical protein	Accessory	Y	Y	1	2	sortaseA	LPXTG
1364	131002_00219	hypothetical protein	Accessory	Y	Y	1	1	sortaseD	LPXTG
1365	131002_01170	hypothetical protein	Accessory	Y	Y	1	2	sortaseD	LPXTG
1366	131002_01957	Serine-aspartate repeat-containing protein D precursor	Accessory	Y	Y	1	1	sortaseD	LPXTG
1367	131002_02164	hypothetical protein	Accessory	Y	Y	1	2	sortaseD	LPXTG

Abbreviations:

Amb	Ambiguous
Sec-SpI	Secreted protein with SpI signal peptide
Sec-Lipo	Lipoprotein
TM	Protein with transmembrane domains
TM-Sec	Transmembrane proteins with SpI signal peptide
TM-Sec-Lipo	Transmembrane proteins with SpII signal peptide (Lipobox)
LPXTG	Cell wall-anchored proteins
