# Supplementary material for: Genomic analyses reveal two distinct lineages of Corynebacterium ulcerans strains
Source: New Microbes New Infect. 2018 May 25;25:7–13. doi: 10.1016/j.nmni.2018.05.005 (PMC6038270; doi:10.1016/j.nmni.2018.05.005)
Supplement: Multimedia component 3 [file mmc3.pdf]

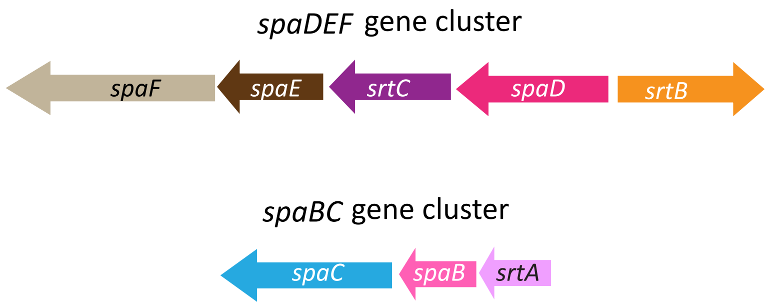

Supplementary Figure 1. A general organisation of pilus gene clusters in *C. ulcerans* strains. The direction of the arrow indicates the orientation of the coding sequence. The schematic is not to scale.
